# Integrative omics analysis elucidates the genetic basis underlying seed weight and oil content in soybean

**DOI:** 10.1093/plcell/koae062

**Published:** 2024-02-27

**Authors:** Xiaobo Yuan, Xinyu Jiang, Mengzhu Zhang, Longfei Wang, Wu Jiao, Huatao Chen, Junrong Mao, Wenxue Ye, Qingxin Song

**Affiliations:** State Key Laboratory of Crop Genetics & Germplasm Enhancement and Utilization, Jiangsu Collaborative Innovation Center for Modern Crop Production, Nanjing Agricultural University, No. 1 Weigang, Nanjing, Jiangsu 210095, China; State Key Laboratory of Crop Genetics & Germplasm Enhancement and Utilization, Jiangsu Collaborative Innovation Center for Modern Crop Production, Nanjing Agricultural University, No. 1 Weigang, Nanjing, Jiangsu 210095, China; State Key Laboratory of Crop Genetics & Germplasm Enhancement and Utilization, Jiangsu Collaborative Innovation Center for Modern Crop Production, Nanjing Agricultural University, No. 1 Weigang, Nanjing, Jiangsu 210095, China; State Key Laboratory of Crop Genetics & Germplasm Enhancement and Utilization, Jiangsu Collaborative Innovation Center for Modern Crop Production, Nanjing Agricultural University, No. 1 Weigang, Nanjing, Jiangsu 210095, China; State Key Laboratory of Crop Genetics & Germplasm Enhancement and Utilization, Jiangsu Collaborative Innovation Center for Modern Crop Production, Nanjing Agricultural University, No. 1 Weigang, Nanjing, Jiangsu 210095, China; Institute of Industrial Crops, Jiangsu Academy of Agricultural Sciences, No. 50 Zhongling, Nanjing, Jiangsu 210014, China; State Key Laboratory of Crop Genetics & Germplasm Enhancement and Utilization, Jiangsu Collaborative Innovation Center for Modern Crop Production, Nanjing Agricultural University, No. 1 Weigang, Nanjing, Jiangsu 210095, China; State Key Laboratory of Crop Genetics & Germplasm Enhancement and Utilization, Jiangsu Collaborative Innovation Center for Modern Crop Production, Nanjing Agricultural University, No. 1 Weigang, Nanjing, Jiangsu 210095, China; State Key Laboratory of Crop Genetics & Germplasm Enhancement and Utilization, Jiangsu Collaborative Innovation Center for Modern Crop Production, Nanjing Agricultural University, No. 1 Weigang, Nanjing, Jiangsu 210095, China

## Abstract

Synergistic optimization of key agronomic traits by traditional breeding has dramatically enhanced crop productivity in the past decades. However, the genetic basis underlying coordinated regulation of yield- and quality-related traits remains poorly understood. Here, we dissected the genetic architectures of seed weight and oil content by combining genome-wide association studies (GWAS) and transcriptome-wide association studies (TWAS) using 421 soybean (*Glycine max*) accessions. We identified 26 and 33 genetic loci significantly associated with seed weight and oil content by GWAS, respectively, and detected 5,276 expression quantitative trait loci (eQTLs) regulating expression of 3,347 genes based on population transcriptomes. Interestingly, a gene module (IC79), regulated by two eQTL hotspots, exhibited significant correlation with both seed weigh and oil content. Twenty-two candidate causal genes for seed traits were further prioritized by TWAS, including *Regulator of Weight and Oil of Seed 1* (*GmRWOS1*), which encodes a sodium pump protein. GmRWOS1 was verified to pleiotropically regulate seed weight and oil content by gene knockout and overexpression. Notably, allelic variations of *GmRWOS1* were strongly selected during domestication of soybean. This study uncovers the genetic basis and network underlying regulation of seed weight and oil content in soybean and provides a valuable resource for improving soybean yield and quality by molecular breeding.

## Teaser

Integration of multiple association studies reveals genetic loci, coexpression modules, and candidate genes that are significantly associated with simultaneous regulation of seed weight and oil content.

## Introduction

In flowering plants, seeds accumulate large amounts of storage compounds such as oil, carbohydrate, and protein in the embryo and/or in the extra-embryonic tissues. Seed storage reserves not only affect the seed quality and viability but also supply the requirement of human and animal diets (Li et al. 2019). Soybean (*Glycine max* L. Merr.) is one of the most valuable oilseed crops with high global economic relevance, accounting for 60% of the world oilseed production in 2021/2022 (www.statista.com). Commodity soybeans contain approximately 20% oil with a favorable proportion of linoleic acid (Fang et al. 2017). Oil is accumulated rapidly in soybean seeds during the pod-filling stage, which is accompanied by a dramatic increase in seed size (Song et al. 2013).

Modern cultivated soybean is generally believed to have been domesticated from the wild progenitor *Glycine soja* in China 6,000 to 9,000 years ago, through dramatic changes in morphological and physiological traits, including loss of pod shattering and reduced seed dormancy (Kim et al. 2012; Zhou et al. 2015). Although wide variations in the seed weight and oil content are observed among soybean accessions, cultivated soybeans generally produce larger seeds with higher oil content than wild soybeans (Kim et al. 2012). Seed weight and oil content are two of the most important determinants of soybean yield and quality, which are highly associated with each other (Goettel et al. 2022). In general, seed weight is positively associated with oil content, although negative association is also reported in a few soybean germplasms (Bheemanahalli et al. 2022). Decoding the genetic and molecular basis underlying synergistic regulation of seed weight and oil content during soybean domestication and improvement is indispensable for developing high-yield and high-quality varieties in soybean.

To date, over 300 quantitative trait loci (QTLs) controlling seed weight and oil content have been identified using linkage analysis and genome-wide association studies (GWAS) in soybean (SoyBase, https://soybase.org/). Several proteins are known be involved in regulation of the seed traits, including Phosphatase 2C-1 (GmPP2C-1) and GmKIX8-1 for seed weight (Lu et al. 2017; Nguyen et al. 2021), GmOLEO1 and GmZF351 for oil content (Li et al. 2017; Zhang et al. 2019). Especially, some proteins have pleiotropic functions in multiple seed phenotypes (seed weight, oil, and protein content), such as Protein, Oil, Weight Regulator 1 (POWR1), GmSWEET10a/b, and Seed Thickness 05 (GmST05) (Wang et al. 2020; Duan et al. 2022; Goettel et al. 2022). However, the genetic mechanism and causal genes underlying QTLs regulating seed weight and oil content remain largely unknown. With the development of multiomics technologies, the integration of GWAS and transcriptome-wide association studies (TWAS) has been used to dissect regulatory networks and key regulators that orchestrate important agronomic phenotypes in cotton and rapeseed (Li et al. 2020; Tang et al. 2021; Zhang et al. 2022). Application of multiomics analyses could facilitate the identification of more genes related to seed traits in soybean.

In this study, we identified a number of genetic loci, coexpression modules and candidate genes that were significantly associated with seed weight and oil content by integration of GWAS, expression quantitative trait locus (eQTL) information and TWAS in soybean. A key gene under strong artificial selection was verified to simultaneously regulate seed weight and oil content using gene overexpression and CRISPR/Cas9-mediated knockout. These findings shed light on the genetic basis for synergistic regulation of seed weight and oil content and provide a valuable resource for the breeding of high-yield and high-quality varieties in soybean.

## Results

### Identification of genomic loci controlling seed weight and oil content

**Figure 1.**
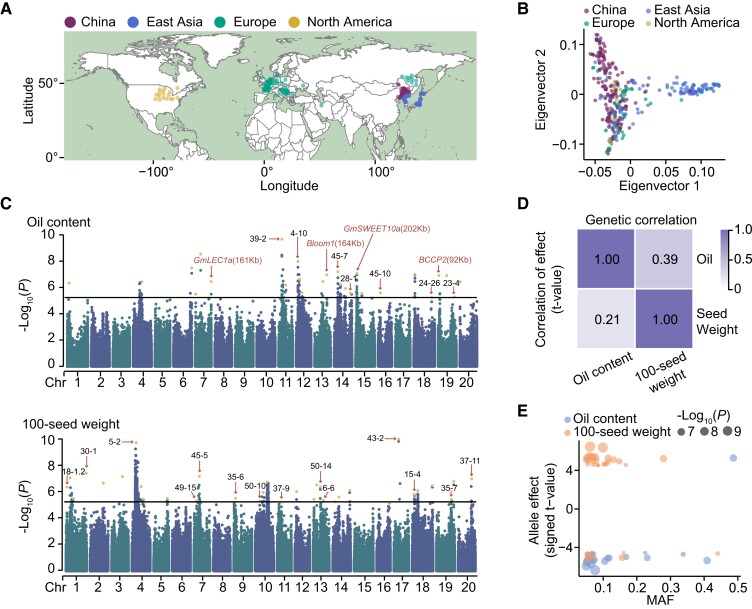
GWAS of 100-seed weight and oil content in soybean. **A)** The geographic distribution of 421 soybean accessions. **B)** PCA plot of the first two principal components of soybean accessions. **C)** Manhattan plots of GWAS for 100-seed weight (bottom panel) and oil content (top panel). The horizontal solid lines indicate the significance threshold of GWAS (−log_10_(*P*) = 5.20). The yellow dots represent the lead SNPs after LD-based result clumping. The number in brackets indicates the distance between a gene and its closest lead SNP. The known QTLs related with 100-seed weight and oil content that overlap GWAS peaks are labeled using names assigned by SoyBase in black font. **D)** Heatmap describing the genetic correlation between 100-seed weight and oil content. The value in upper right square was calculated by bivariate GREML analysis, and the value in lower left square represents the correlation of variant effect (signed t-value). **E)** The relationship between MAF and effect size of significant variant (*P* < 6.24 × 10^−6^) for 100-seed weight (orange) and oil content (blue). MAF, minor allele frequency.

To investigate the genetic basis for yield and quality traits in soybean, we collected 421 cultivated accessions from 24 countries in four main regions with high latitude, namely China, East Asia, North America, and Europe (Fig. 1A; Supplementary Data Set 1). A total of 19 billion paired-end reads were generated by resequencing with an average depth of 7× for each accession (Supplementary Data Set 2). We aligned the sequencing data to a reference genome (Williams 82) and obtained 6,063,109 single-nucleotide polymorphism (SNPs) and 1,097,715 InDels. A principal component analysis (PCA) using variants after LD pruning suggested that accessions in East Asia were the most genetically distant from other regions, and for accessions in China, North America, and Europe, there were more genetic variations within sub-populations than between sub-populations (Fig. 1B; Supplementary Fig. S1A).

The yield-related trait 100-seed weight and quality-related trait oil content were measured in Harbin in 2019 and 2020. Significant correlations in seed oil content (0.617) and 100-seed weight (0.883) were observed among these accessions across two independent years (Supplementary Fig. S1B). Then, the best linear unbiased estimator (BLUE) value for each accession was further calculated for each trait. The accessions collected from North America exhibited the highest oil content, while accessions from East Asia showed the lowest oil content, but highest 100-seed weight (Supplementary Fig. S1C). The lowest median value of oil content in accessions from East Asia was due to a higher percentage of accessions with lower oil content (Supplementary Fig. S1D). GWAS was performed using a mixed linear model (MLM) to determine key genetic loci underlying oil content and seed weight (Fig. 1C; Supplementary Fig. S1, E and F). A total of 26 and 33 significantly associated loci were detected using a threshold of 6.24 × 10^−6^ for oil content and 100-seed weight, respectively (Fig. 1C; Supplementary Data Set 3). It is worth noting that about 40% of loci were colocalized with the reported QTLs related to seed weight and oil content in the SoyBase database (Fig. 1C). Moreover, four known genes related to oil content were colocalized with the significantly associated loci, including *LEAFY COTYLEDON 1a* (*GmLEC1a*), *Bloom1* (*B1*), *GmSWEET10a*, and *Biotin Carboxyl Carrier Protein 2* (*BCCP2*) (Li et al. 2017; Zhang et al. 2017, 2018; Wang et al. 2020), suggesting the reliability of phenotypic data and effectiveness of association analyses (Fig. 1C). A significant correlation (0.23) between seed oil content and 100-seed weight was observed among 421 accessions (Supplementary Fig. S1D). Consistently, bivariate genome-based restricted maximum likelihood (GREML) analyses revealed the significant positive genetic correlation between seed weight and oil content (*r*_g_ = 0.39, standard error = 0.098) (Fig. 1D). In addition, effect estimates for SNP associations with seed weight were positively correlated with those related to oil content in GWAS analyses (*r*_g_ = 0.21, *P* < 2.2e^−16^) (Fig. 1D). These results indicate the potential of a genetic basis for improving both seed weight and oil content simultaneously (Fig. 1D). To dissect the nature of the heritable contribution to seed traits, we further deciphered the genetic architectures for seed weight and oil content which depend on the numbers and frequencies of genetic variants, the magnitude of their effects and their interactions with each other and the environment (Timpson et al. 2018). Interestingly, we found the minor alleles that exceeded the significant threshold mainly showed a negative effect for oil content, whereas most minor alleles exerted a positive influence on seed weight (Fig. 1E). Similar patterns were obtained based on the published population containing 2,898 accessions (Supplementary Fig. S1G).

### eQTL mapping and hotspot detection

To explore the effect of genetic variants on gene expression and dissect the causal genes for variations of seed traits, we generated expression patterns of seeds at 21 days after flowering (DAF) for 238 soybean accessions using 3′ RNA-seq and obtained 0.7 billion sequencing reads with an average of 40 million for each accession (Supplementary Data Set 4). Of 52,872 annotated genes in the soybean genome, 25,089 genes expressed in more than 5% of the accessions were used for subsequent analysis (Supplementary Fig. S2A). The top 200 most highly expressed genes accounted for more than 50% of total transcriptions in soybean developing seeds, including known genes related to protein storage [glycinin genes *GLYCININ3-5* (*Gy3-5*)], oil synthesis (*GmOLEO1*, *GmSWEET10b*), seed size [*Cytochrome P450 78A57* (*GmCYP78A57*), *GmST05*], and photosynthesis [*Zeaxanthin Epoxidase* (*ZEP*)] (Beilinson et al. 2002; Zhao et al. 2016; De Souza et al. 2022; Duan et al. 2022) (Supplementary Fig. S2B). Gene ontology (GO) analysis showed that the highly expressed genes were enriched in lipid storage, cellular response to heat, proteolysis, and photosynthesis (Supplementary Fig. S2C).

**Figure 2.**
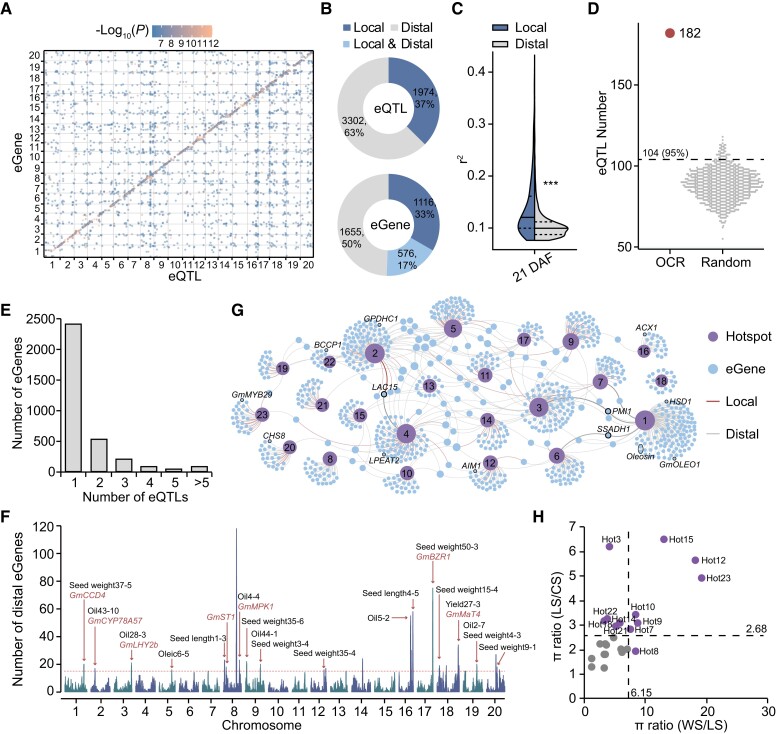
Genome-wide characterization of eQTLs. **A)** Dot plot displaying the associations of eQTLs and their regulated eGenes in 20 chromosomes. The color scale of each dot represents the significance (−log_10_(*P*)) of each eQTL–eGene pair. **B)** The fraction of local and distal eQTLs (top), and the percentage of eGenes regulated by different types of eQTLs (bottom). **C)** Split violin plot showing explained variance (*r*^2^) of local and distal eQTLs for expression of their regulated eGenes. The dashed lines indicate the distribution quantiles (0.25, 0.75). Three asterisks indicate a statistical significance level of *P* < 2.2e^−16^ (two-sided Wilcoxon rank sum test). **D)** More eQTLs in OCRs compared with random genomic regions which were represented by 1,000 times random permutation of OCR positions across the genome. The dashed line indicates 95% confidence interval values. **E)** Counts of eGenes regulated by different numbers of eQTLs. **F)** Number of eGenes regulated by distal eQTLs using 1-Mb windows across the genome. The horizontal dashed line indicates the threshold (15) of eGene numbers regulated by eQTL hotspot regions. The known genes and QTLs related to seed quality and yield are labeled in red and black font, respectively. **G)** Genetic network between eQTL hotspots and eGenes. **H)** Identification of selective hotspots during domestication and improvement. The vertical and horizontal dashed lines indicate the significant genome-wide threshold of selection signals for domestication (≥6.15) and improvement (≥2.68), respectively. Selective hotspots are marked in purple. WS, LS, and CS indicate wild, landrace, or improved soybeans, respectively.

We performed eQTL mapping using a MLM and identified 5,276 eQTLs associated with 3,347 Genes (eGene) (Fig. 2A; Supplementary Data Set 5). Based on the distance (threshold of 1 mb) between eQTLs and eGenes, the eQTLs were divided into 1,974 local eQTLs (37%) and 3,302 distal eQTLs (63%) (Fig. 2B). Correspondingly, 33% (1,116) and 50% (1,655) of eGenes were regulated by local and distal eQTLs, respectively (Fig. 2B). In addition, 17% (576) of eGenes were associated with both local and distal eQTLs (Fig. 2B). Consistent with previous findings in other plants (Wang et al. 2018; Li et al. 2020), the effect of local eQTL on expression variation was larger than that of distal eQTL (*P* < 2.2 × 10^−16^, Wilcoxon rank sum test) (Fig. 2C). The local eQTLs preferred to locate within 10 kb from the transcription start sites (TSSs) of eGenes (Supplementary Fig. S3). Furthermore, we found eQTLs were significantly enriched in open chromatin regions (OCRs) (Fig. 2D), suggesting important role of chromatin accessibility in regulation of gene expression by eQTLs.

Although the majority of eGenes were regulated by only one eQTL, we detected 23 distal eQTL hotspots which could regulate multiple (> 15) eGenes in developing seed (Fig. 2, E and F). Most of these hotspots overlapped with previously reported QTLs and known genes related to seed traits (Fig. 2F), such as *Glycine max carotenoid cleavage dioxygenase 4* (*GmCCD4*) for carotenoid content, *GmMPK1* and *GmMaT4* for isoflavone content, *GmCYP78A57*, *Seed Thickness 1* (*GmST1*), and *GmBZR1* for seed size (Zhao et al. 2016; Lu et al. 2017; Wu et al. 2020; Ahmad et al. 2021; Gao et al. 2021; Li et al. 2022). A total of 711 distal genes and 130 local genes were regulated by eQTL hotspots. GO and Kyoto Encyclopedia of Genes and Genomes (KEGG) enrichment analysis revealed eGenes regulated by distinct eQTL hotspots were enriched in different terms (Supplementary Fig. S4). For example, genes regulated by hotspot1 (Hot1) were enriched in lipid storage, whereas genes regulated by hotspot2 (Hot2) were involved in regulation of hydrolase activity, defense response, and secondary metabolic process (Supplementary Fig. S4). The genes regulated by 23 hotspots could form a genetic network through eQTL-eGene connection (Fig. 2G). Consistent with a distinct biological function for each hotspot, most genes were regulated by only one hotspot (Fig. 2G). Notably, several essential enzyme genes were regulated by multiple hotspots, including *SUCCINIC SEMIALDEHYDE DEHYDROGENASE 1* (*SSADH1*) in the γ-aminobutyrate (GABA) shunt of tricarboxylic acid cycle pathway, *PLASTID MOVEMENT IMPAIRED 1* (*PMI1*) in L-ascorbate synthesis and *LACCASE-LIKE 15* (*LAC15*) in lignin and flavonoids biosynthesis (Bouché et al. 2003; Pourcel et al. 2005; Liang et al. 2006) (Fig. 2G). Based on published resequencing data for wild and cultivated soybeans including landraces and cultivars (Liu et al. 2020), we found 12 eQTL hotspots experienced strong artificial selection during domestication or improvement of soybean (Fig. 2H).

### Gene modules involved in coordinated regulation of seed weight and oil content

**Figure 3.**
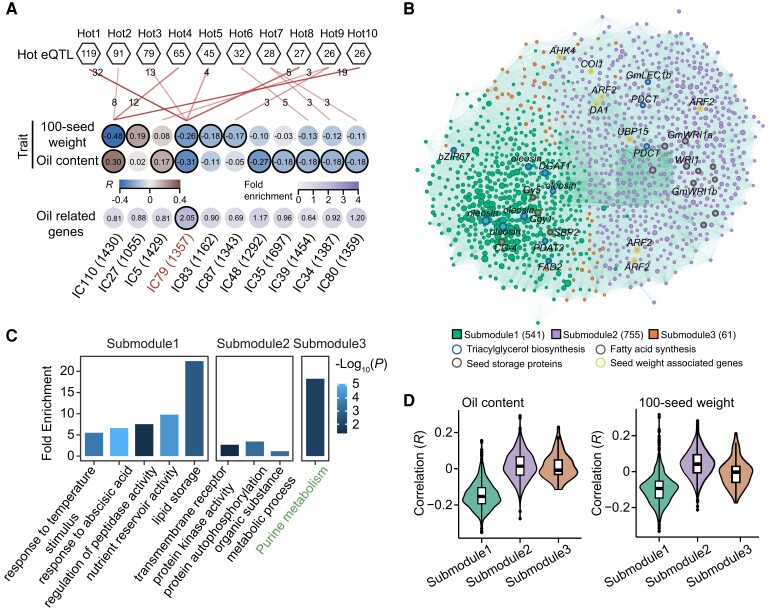
Genetic network of module IC79 involved in regulation of seed weight and oil content. **A)** Identification of coexpression modules significantly associated with 100-seed weight and oil content. The first two rows of the heatmap represent correlations between modules and seed traits, and the last row indicates the fold enrichment of known trait-related genes. The thickness of red solid line indicates the enrichment of genes distally regulated by the eQTL hotspot in each module. The number of genes regulated by each eQTL hotspot is marked on each line. **B)** Correlation network of genes in module IC79. The genes related to seed storage proteins, seed weight, and lipid synthesis are labeled in the network. **C)** Overrepresented GO (black) and KEGG (green) terms for genes in three submodules. **D)** Correlation of genes in different submodules with 100-seed weight (right) and oil content (left). In each box plot, borders represent the first and third quartiles, center line denotes median, and whiskers extend to 1.5 times the interquartile range beyond the quartiles.

To provide a view of phenotype variations arising from coordinated gene regulation, we inferred a gene–gene coexpression network by independent component analysis (ICA). Of the 113 coexpression modules constructed, 8 and 5 modules were significantly correlated with oil content and 100-seed weight, respectively (Fig. 3A). Interestingly, these modules contained multiple genes distally regulated by eQTL hotspots (Fig. 3A). For 23 eQTL hotspots, we selected the ten hotspots with the highest number of distally regulated genes for analysis. The other eQTL hotspots regulated fewer genes and had a limited relationship with these modules. Two modules (IC79 and IC110) showed significant correlation with both 100-seed weight and oil content, whereas only IC79 (1,357 genes) exhibited identical direction of associations with both traits (Fig. 3A). In addition, genes related to oil metabolism were significantly enriched in the module IC79 (Fig. 3A, Supplementary Data Set 6). Therefore, we further analyzed the function of module IC110 and IC79 in detail (Supplementary Data Sets 7 and 8).

Based on the expression correlation among genes, module IC79 was divided into three submodules, of which submodule1 (541 genes) was enriched in the GO terms for lipid storage, nutrient reservoir activity, regulation of peptidase activity, response to abscisic acid, and temperature stimulus; submodule2 (755 genes) was relevant to protein autophosphorylation, transmembrane receptor protein kinase activity, and organic substance metabolic process; and submodule3 (61) was mainly enriched in KEGG pathway for purine metabolism (Fig. 3, B and C). Lipid synthesis includes fatty acid synthesis, triacylglycerol synthesis, and oil body formation (Murphy 1990; Baud and Lepiniec 2010). Lipid is mainly stored as triacylglycerol in the oil body of seed cells. Notably, submodule1 and submodule2 contained many genes involved in oil synthesis, such as acyltransferase genes [*DIACYLGLYCEROL ACYLTRANSFERASE 1* (*DGAT1*) and *PHOSPHOLIPID:DIACYLGLYCEROL ACYLTRANSFERASE 2* (*PDAT2*)], transcription factor genes [*GmbZIP67*, *WRINKLED 1a/b* (*GmWRI1a/b*)], and oil body oleosin genes (Zhang et al. 2009; Mendes et al. 2013; Zhang et al. 2017). AUXIN RESPONSE FACTOR 2 (ARF2) and DA1 are negative regulators of seed size in Arabidopsis (*Arabidopsis thaliana*) (Schruff et al. 2006; Xia et al. 2013). Soybean homologous genes of *ARF2* and *DA1* were detected in submodule2 (Fig. 3B).

**Figure 4.**
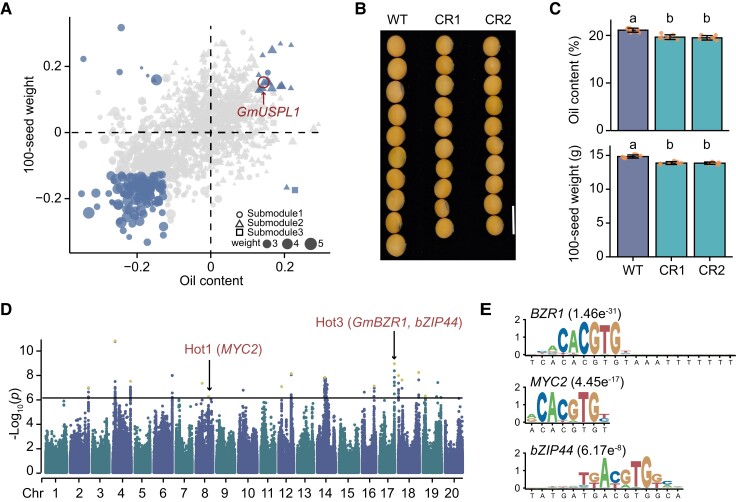
Identification of genes significantly associated with both seed weight and oil content. **A)** Scatter plot showing correlation of genes in submodule1 and submodule2 with 100-seed weight (*y* axis) and oil content (*x* axis). The genes significantly associated with both 100-seed weight and oil content are marked in blue (*P* < 0.01, Pearson correlation). **B)** Representative seeds of wild-type plants and *Gmuspl1* mutants (CR1 and CR2). Scale bar, 1 cm. **C)** Seed oil content (upper) and 100-seed weight (bottom) in wild type and *Gmuspl1* mutants. The data were shown as mean ± SD (*n* = 6). Different letters denote significant differences (*P* < 0.05) from two-tailed t-test. The value of each replicate is represented by a dot. **D)** Manhattan plot of GWAS using the expression pattern of IC79 as a phenotype. The horizontal solid line indicates the significance threshold of GWAS (−log_10_(*P*) = 6.11). The yellow dots represent the lead SNPs after LD-based result clumping. **E)** Enrichment of *BZR*, *MYC2*, and *bZIP44* motif in the promoter sequences of genes in IC79.

We calculated correlations between expression and seed traits for each gene in module IC79. Negative correlations between expression and seed traits were observed in most genes in submodule1, while genes in submodule2 and 3 were mainly positively correlated with 100-seed weight and oil content (Fig. 3D). We found 69 genes showing significant correlations with both 100-seed weight and oil content at 21 DAF in module IC79 (Fig. 4A). To further explore the genetic basis for synergistic regulation of seed weight and oil content, we analyzed the correlations between these genes and seed traits at different seed developmental stages (4, 6, and 8 weeks after flowering) using published datasets (Liu et al. 2020). We found 18 genes were concurrently associated with both traits in seeds at different developmental stages (Supplementary Fig. S5), such as Glyma.19G004800 for oleosin synthesis and Glyma.10G297600 for lipid transport. Among the 69 genes, one gene (Glyma.11G119100) showed a positive correlation with both seed traits and was colocalized with loci significantly associated with oil content in GWAS as well (Supplementary Data Set 3). Three major haplotypes of Glyma.11G119100 were identified, of which 314 accessions harbored Hap 1, 49 accessions harbored Hap 2, and 30 accessions harbored Hap 3 (Supplementary Fig. S6A). Accessions with Hap 3 showed lower oil content and reduced expression of Glyma.11G119100 compared to accessions harboring Hap 1/2 (Supplementary Fig. S6B). Glyma.11G119100 was predicted to encode a BURP domain protein targeted to the protein storage vacuoles and shared 39.1% protein sequence identity with UNKNOWN SEED PROTEIN LIKE 1 (AtUSPL1) in Arabidopsis. Therefore, Glyma.11G119100 was designated as *GmUSPL1*. In Arabidopsis, overexpression of *AtUSPL1* affected seed development, protein storage vacuoles and lipid vesicles morphology (Van Son et al. 2009). To validate the function of GmUSPL1 in regulation of seed weight and oil content, we knocked out *GmUSPL1* in Williams 82 by the CRISPR/Cas9 system and obtained two homozygous knockout lines (CR1 and CR2) (Supplementary Fig. S6C). Compared with wild type, CR1 and CR2 exhibited a significant decrease in 100-seed weight, seed length, seed width, and oil content (Fig. 4, B and C; Supplementary Fig. S6, D and E). Meanwhile, there were no obvious variations in plant architecture and seed number per plant between wild and knockout lines (Supplementary Fig. S6, F and G).

To detect genomic loci tending to distally regulate the module IC79, we performed GWAS using the expression pattern of module IC79 as a phenotype. We identified 14 genomic loci significantly associated with module IC79, of which 2 genomic loci were colocalized with eQTL hotspots (hotspot1 and hotspot3) (Fig. 4D; Supplementary Table S1). All 32 eGenes in module IC79 regulated by hotspot1 belonged to submodule1, and of the 15 eGenes regulated by hotspot3, 12 and 3 genes belonged to submodule1 and submodule2, respectively. In addition, the genes regulated by hotspot1 and hotspot3 had higher module weights and higher correlation degree with seed traits compared with the remaining genes in IC79 (Supplementary Fig. S7A to C). To prioritize the candidate key regulators controlling the expression pattern of module IC79 in GWAS loci, the promoter regions of genes in module IC79 were extracted for enrichment analysis of binding motifs of transcription factors (TFs). Interestingly, we found binding motifs of *MYC2* in hotspot1 and *bZIP44* and *BZR1* in hotspot3 were significantly enriched in promoters of genes in IC79 (Fig. 4E). BZR1 and MYC2 were reported to be involved in regulation of seed weight in Arabidopsis and soybean in recent studies (Lu et al. 2017; Hu et al. 2021; Wei et al. 2023). There were significant differences in 100-seed weight, oil content, and IC79 expression levels among accessions with distinct haplotypes for *MYC2* (Hap1 vs Hap2/3) and *bZIP44* (Hap1 vs Hap2) (Supplementary Fig. S8). Therefore, MYC2 and bZIP44 may be the potential regulators of module IC79.

In addition to module IC79, module IC110 could be subdivided into three submodules. In submodule 1, genes were positively correlated with oil content and negatively correlated with 100-seed weight, while the expression patterns of genes in submodules 2 and 3 were in contrast to submodule 1 (Supplementary Fig. S9, A and B). Furthermore, we found 30 genes showing opposite correlations with 100-seed weight and oil content (*P* < 0.01), including *FRUCTOSE-BISPHOSPHATE ALDOLASE 2* (*FBA2*) and *PECTIN METHYLESTERASE 47* (*PME47*) (Supplementary Fig. S9C). Consistent with this notion, *PME47* was reported to be involved in regulation of seed weight, and *FBA2* was recognized as a candidate gene for oil content in previous studies (Du et al. 2017; Kumar et al. 2022).

### Identification of key genes regulating seed weight and oil content by TWAS

**Figure 5.**
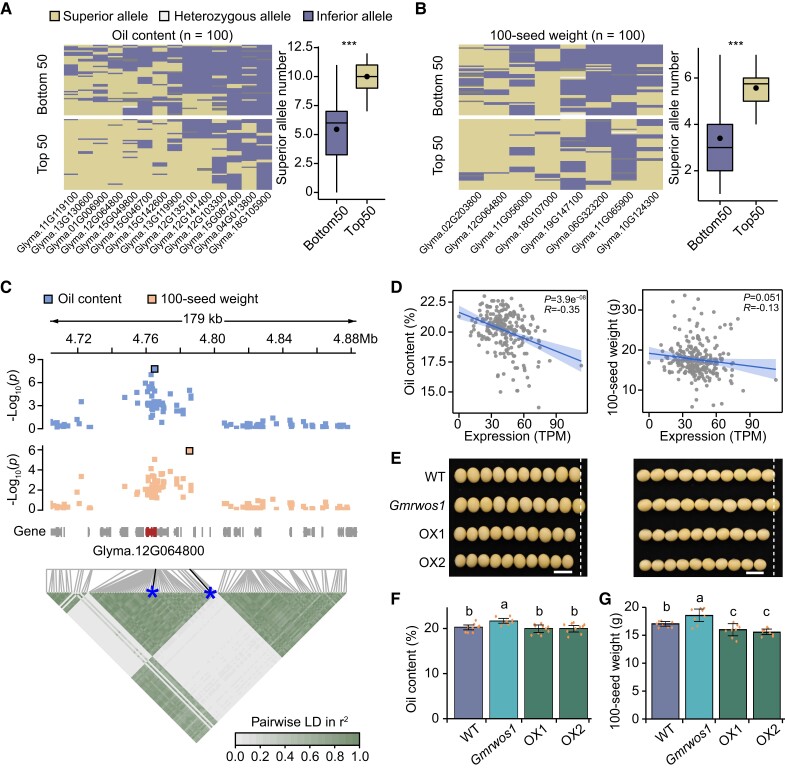
Regulation of seed weight and oil content by *GmRWOS1*. **A** and **B)** The aggregation of superior alleles in the top and bottom 50 accessions with highest and lowest oil content **(A)** or 100-seed weight **(B)** in the soybean population. In each box plot (*n* = 50), borders represent the first and third quartiles, center line denotes median, and whiskers extend to 1.5 times the interquartile range beyond the quartiles. *** indicates *P* < 0.001 (Wilcoxon rank sum test). **C)** Local Manhattan plot (top) and linkage disequilibrium plot (bottom) for SNPs surrounding the peak on chromosome 12. Asterisks indicate positions of the two lead SNPs. **D)** Correlation of *GmRWOS1* expression with 100-seed weight (right) and oil content (left). Each dot indicates one soybean accession. **E)** The representative seeds of wild type, *Gmrwos1* mutant, and overexpression lines OX1/2. Scale bars, 1 cm. **F** and **G)** Seed oil content **(F)** and 100-seed weight **(G)** in wild type, *Gmrwos1* mutant and overexpression lines OX1/2. The data were shown as mean ± SD (*n* = 8 or 10). The value of each replicate is represented by a dot. Different letters denote significant differences (*P* < 0.05) from two-tailed t-test.

To pinpoint causal genes for GWAS loci, we performed TWAS to identify genes whose expression levels were significantly associated with seed weight and oil content. In total, 8 and 14 candidate regulatory genes were detected for seed weight and oil content, respectively (Table 1). It was worth noting that 50% (7/14) and 37.5% (3/8) of genes identified by TWAS were located in significantly associated loci detected by GWAS (Supplementary Data Set 3). To evaluate contributions of superior alleles of these candidate genes to seed traits, we selected the top 50 and bottom 50 accessions according to seed traits from soybean population, and assessed the aggregation of superior alleles. A significant divergency in the number of superior alleles was observed between the top and bottom 50 accessions (Fig. 5, A and B). The soybean accessions exhibiting higher seed weight or oil content contained a greater number of superior alleles (Fig. 5, A and B). In addition, we observed similar results using another published soybean population with 2,898 accessions (Supplementary Fig. S10, A and B) (Liu et al. 2020), indicating substantial contributions of these candidate genes to regulation of seed weight and oil content during soybean improvement. Strikingly, a gene (Glyma.12G064800) encoding a K-stimulated pyrophosphate-energized sodium pump protein was identified as the candidate gene controlling both seed weight and oil content (Table 1). Glyma.12G064800 located within a genomic locus of chromosome 12 which was significantly associated with both seed weight and oil content in GWAS analysis. The lead SNPs for the two traits were located in the same 50 kb linkage block (Fig. 5C). Indeed, there was a significant negative correlation between oil content and expression of Glyma.12G064800 (Fig. 5D). Meanwhile, this gene also showed a negative correlation with 100-seed weight, although the correlation was not statistically significant (Fig. 5D). These results suggest Glyma.12G064800 may be the causal gene for the GWAS locus and was designated as *Regulator of Weight and Oil of Seed 1* in soybean (*GmRWOS1*).

**Table 1.** The candidate genes for seed weight and oil content identified by TWAS

Trait	Gene	Lead GWAS SNP	GWAS *P*-value	TWAS Z-score	TWAS *P*-value	Annotation
Oil	Glyma.01G006900	Gm01:662815	1.48e^−04^	3.08	2.07e^−03^	Unknown function
Oil	Glyma.04G013800	Gm04:1012135	3.43e^−04^	3.58	3.43e^−04^	Jasmonate-zim-domain protein 1
Oil	Glyma.11G119100	Gm11:9122858	2.15e^−10^	3.19	1.41e^−03^	Unknown seed protein like 1
Oil	Glyma.12G064800	Gm12:4765210	1.08e^−08^	−3.83	1.28e^−04^	K-stimulated pyrophosphate-energized sodium pump protein
Oil	Glyma.12G103300	Gm12:8930945	2.29e^−07^	−4.06	4.87e^−05^	S-adenosyl-L-methionine-dependent methyltransferase
Oil	Glyma.12G135100	Gm12:15761610	2.49e^−06^	3.79	1.52e^−04^	Translation initiation factor SUI1 family protein
Oil	Glyma.12G141400	Gm12:17587181	1.27e^−05^	−3.52	4.36e^−04^	Cytokine-induced anti-apoptosis inhibitor 1, Fe-S biogenesis
Oil	Glyma.13G119900	Gm13:22203025	4.35e^−05^	3.23	1.25e^−03^	Novel plant snare 13
Oil	Glyma.13G130600	Gm13:23114123	3.55e^−07^	3.38	7.21e^−04^	Unknown function
Oil	Glyma.15G046700	Gm15:3658418	8.72e^−08^	4.35	1.34e^−05^	Maternal effect embryo arrest 9
Oil	Glyma.15G049800	Gm15:3658418	8.72e^−08^	−3.29	1.01e^−03^	Nodulin MtN21/EamA-like transporter family protein
Oil	Glyma.15G087400	Gm15:6771913	8.16e^−06^	−3.37	7.52e^−04^	Putative adipose-regulatory protein
Oil	Glyma.15G142600	Gm15:11253117	1.57e^−05^	3.24	1.18e^−03^	WD40/YVTN repeat-like-containing domain
Oil	Glyma.18G105900	Gm18:11849686	4.31e^−05^	3.57	3.61e^−04^	Galacturonosyltransferase 15
SW	Glyma.02G203800	Gm02:40313191	9.32e^−05^	3.16	1.57e^−03^	WRKY DNA-binding protein 32
SW	Glyma.06G323200	Gm06:50559402	8.46e^−05^	−3.02	2.54e^−03^	Unknown function
SW	Glyma.10G124300	Gm10:32983684	1.78e^−07^	−3.67	2.39e^−04^	Ankyrin repeat family protein
SW	Glyma.11G056000	Gm11:4099254	6.17e^−05^	3.55	3.92e^−04^	K-stimulated pyrophosphate-energized sodium pump protein
SW	Glyma.11G065900	Gm11:5054063	8.49e^−06^	3.69	2.20e^−04^	Protein phosphatase 2C family protein
SW	Glyma.12G064800	Gm12:4785599	9.58e^−07^	−3.36	7.92e^−04^	K-stimulated pyrophosphate-energized sodium pump protein
SW	Glyma.18G107000	Gm18:12273811	5.07e^−06^	−3.26	1.10e^−03^	Phosphoribosyltransferase family protein
SW	Glyma.19G147100	Gm19:40884584	3.69e^−06^	3.53	4.18e^−04^	KH domain-containing protein/zinc finger family protein

Oil, oil content; SW, 100-seed weight.

To further confirm the function of GmRWOS1 in the regulation of seed weight and oil content in soybean, we disrupted *GmRWOS1* in Williams 82 via the CRISPR/Cas9 genome editing system with a guide RNA targeting the third exon of *GmRWOS1* (Supplementary Table S2). We generated a homozygous loss-of-function mutant line named as *Gmrwos1*, harboring a frameshift mutation produced by a 4-bp deletion in the target site (Supplementary Fig. S11A). Significant increases of 100-seed weight (+8.80%), grain length (+2.60%), grain width (+3.14%), and oil content (+6.92%) were observed in *Gmrwos1* compared with wild type (Fig. 5E to G; Supplementary Fig. S11, B and C). Plant architecture and seed number per plant were similar between the wild type and *Gmrwos1* (Supplementary Fig. S11, D and E).

Meanwhile, we overexpressed *GmRWOS1* driven by the 35S promoter in Williams 82 and obtained two independent transgenic lines (OX1 and OX2). The RT-qPCR results revealed that the expression levels of *GmRWOS1* were significantly increased in overexpression lines OX1/2 compared with wild type (Supplementary Fig. S11F; Supplementary Table S2). Phenotypic analysis showed that overexpression transgenic lines OX1/2 exhibited a significant decrease of seed weight compared to the wild type (Fig. 5, E and G). A decrease of oil content was also observed in OX1/2 although it was not significant (Fig. 5F). There were no significant variations in plant architecture and seed number per plant between OX1/2 and wild type (Supplementary Fig. S11, D and E). These findings suggest that GmRWOS1 negatively regulates seed weight and oil content in soybean.

### Selection of *GmRWOS1* during soybean domestication

**Figure 6.**
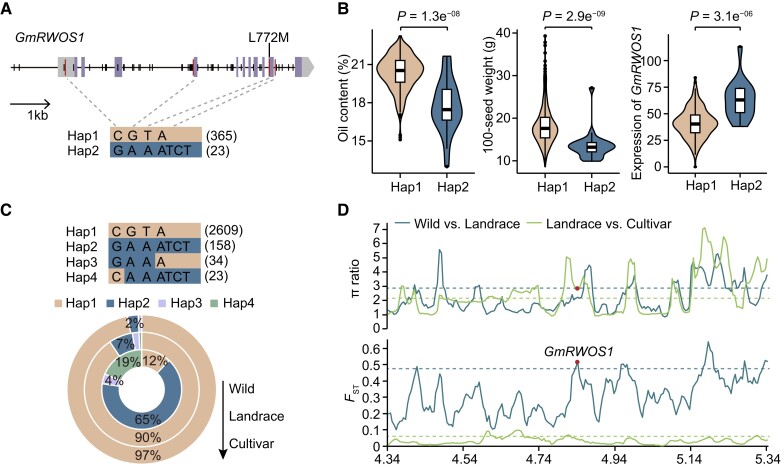
Distribution and diversity analysis of *GmRWOS1* alleles in soybean. **A)** The spectrum of two haplotypes (Hap1 and Hap2) for *GmRWOS1* in 421 soybean accessions. The digits in brackets display the accession number with each haplotype. Vertical lines represent variants and the most significant four variants are marked in red. **B)** Differences of oil content, 100-seed weight and *GmRWOS1* expression between individuals with different haplotypes. In each box plot, borders represent the first and third quartiles, center line denotes median, and whiskers extend to 1.5 times the interquartile range beyond the quartiles. The statistical significance was calculated by Wilcoxon rank sum test. **C)** Distribution of four haplotypes in 2,898 previously resequenced accessions and their proportions in wild soybeans, landraces, and cultivars. Inner to outer circles indicate wild soybeans, landraces and cultivars. **D)** The π ratio and *F*_ST_ values of flanking region at *GmRWOS1* in wild soybeans, landraces, and cultivars. The horizontal dashed lines indicate the genome-wide thresholds of wild soybeans vs landraces and landraces vs cultivars (top 10%). *GmRWOS1* is labeled with a red dot.

Two major haplotypes (Hap1and Hap2) of *GmRWOS1* were identified in 421 accessions, of which 365 accessions harbored Hap1 and 23 accessions harbored Hap2 (Fig. 6A). Accessions containing Hap1 exhibited lower expression of *GmRWOS1*, higher oil content and higher 100-seed weight than accessions harboring Hap 2 (Fig. 6B).

Cultivated soybean was domesticated from wild soybean, accompanied with increased seed weight and oil content. Using a SNP dataset from 2,898 previously resequenced accessions, we detected four major haplotypes (Hap1–4) (Fig. 6C). The frequency of Hap1 was dramatically increased from wild soybean to landraces and continuously raised in cultivars (Fig. 6C). We further estimated nucleotide diversity (π) and fixation index (*F*_ST_) across the 500 kb genomic region flanking the *GmRWOS1* gene among wild soybeans, landraces, and cultivars. The *F*_ST_ value (0.52) across the genomic region flanking *GmRWOS1* reached the threshold of 0.48 (top 10%) using whole-genome scale evaluation (Fig. 6D), although π ratio (2.25) between wild soybeans and landraces was below the threshold of 2.83 (top 10%). Regarding improvement, the π ratio of landraces relative to cultivars exceeded the cutoff of 2.14 (top 10%) (Fig. 6D). These results suggest *GmRWOS1* underwent strong artificial selection during domestication and improvement of soybean.

## Discussion

Soybean is one of the most important commercial crops worldwide and represents an indispensable source of edible oil for humans. As global demand of vegetable oil and biodiesel is growing, it is imperative for the soybean research community to develop high-yield and high-oil varieties to meet market needs through integration of genomics approaches with traditional breeding. Understanding the regulatory network and key genes for yield-related and oil-related traits could provide the foundation for soybean breeding. Although many QTLs controlling seed weight and oil content have been identified in soybean, causal genes were pinpointed for limited QTLs, impeding the marker-assisted selection for seed weight and oil content. In the present study, we dissected coexpression modules, genomic loci, and regulatory genes controlling seed weight and oil content by combination of GWAS, eQTL information, and TWAS.

GWAS is widely used to identify genetic loci controlling agronomic traits in crops. However, the results from GWAS generally cannot directly translate into causal variants as linkage disequilibrium (LD) persists over long distances in plants. By integration of eQTL and TWAS, we identified candidate causal genes in 10 and 4 GWAS loci controlling seed weight and oil content, respectively (Table 1). For GWAS loci wherein no causal genes were pinpointed by TWAS, the genetic variations may cause gene expression changes in seeds at other developmental stages or other tissues to affect seed traits. The transcriptome data from various tissues may contribute to identification of causal genes in more GWAS loci. On the other hand, protein variations but not expression variations also could mediate phenotype changes in GWAS loci. It's still a big challenge to identify causal genes with protein variations in GWAS loci using multiomics approaches.

The effect and frequency of genetic variants are essential to dissect the genetic architecture of complex traits and the relevance to fitness. For example, Type 1 diabetes mellitus is polygenic and related to low-frequency variants with large effects, whereas Type 2 diabetes mellitus is associated with many genetic variants with small effects (Polychronakos and Li 2011; Scott et al. 2017). Negative selection inferred from the negative relationship between effect size and minor allele frequency (MAF) declines the frequency of variants with large deleterious effects (Zeng et al. 2018). A previous study indicated that major human complex traits are consistent with a model of negative selection (Zeng et al. 2018). In this study, the low frequency of inferior alleles with large effects was observed for oil content (Fig. 1E), indicating most deleterious alleles were gradually removed during artificial selection of soybean varieties with high-oil content. It is urgent to expand germplasm resources and increase the genetic diversity to explore new gene resources for improvement of oil content (Zhou et al. 2015). In contrast to oil content, we detected a low frequency for most superior alleles with large effects for seed weight (Fig. 1E). Hence, there are still numerous superior alleles regulating seed size that could be discovered and utilized to further advance soybean seed weight.

We identified 5,276 eQTLs representing the genomic regulation of gene expression in soybean seeds at 21 DAF (Fig. 2A). The transcriptome data generated from more tissues would contribute to identify more regulatory networks and to explore dynamic effects of eQTLs on gene expression. The majority of eQTL hotspots overlapped with known QTLs related to seed traits (Fig. 2F), suggesting the vital roles of the eQTL hotspots on seed development. However, it is difficult to accurately locate functional genomic variants and to decipher their genetic relationship within eQTL hotspots due to long LD decay distances. The larger population and integration of multiomics data such as TF footprints may help to identify the causal genomic variants controlling gene expression and important agronomic traits in hotspots.

A total of 22 genes controlling seed traits were identified by TWAS and 69 genes showed significant correlations with both seed weight and oil content (Fig. 4A and Table 1), of which *GmRWOS1* and *GmUSPL1* were validated to be involved in regulation of seed weight and oil content. These results prove the effectiveness of multiomics approaches to identify candidate genes controlling agronomic traits and provide candidate target genes for soybean molecular breeding although the functions of these genes should be further verified. Although a superior allele of *GmRWOS1* dominates in the improved cultivars (Fig. 6C), knockout of *GmRWOS1* in Williams 82 with the superior allele could further increase seed weight and oil content (Fig. 5, E-G), suggesting the great potential of genome editing of *GmRWOS1* in soybean improvement. *GmRWOS1* encodes a K-stimulated pyrophosphate-energized sodium pump protein. In animals, the sodium pump serves as the main active transport system, generating a membrane potential and sodium gradient that are utilized by ion channels and cotransporters. However, the role of the sodium pump in plants remains unclear. Inappropriate sodium concentrations may result in lower seed mass and seed viability (Kranner and Seal 2013). The molecular mechanisms by which GmRWOS1 controls seed weight and oil content remain to be further studied. In conclusion, we dissected the genetic architecture of seed weight and oil content in soybean and disclosed the causal candidate genes by a combination of multiomics datasets, which advance our understanding of genetic regulation of gene expression in developing seeds and provide a valuable resource for gene pyramiding in soybean breeding.

## Materials and methods

### Plant materials and growth conditions

For phenotype collection in GWAS, the 421 soybean (*Glycine max*) accessions were planted in Harbin in 2019 and 2020 (Supplementary Data Set 1). To generate seed transcriptomes of the soybean population using 3′ RNA-seq (Supplementary Data Set 4), the 238 soybean accessions were planted in Nanjing in 2019. The transgenic plants and the wild type were grown at the experimental station in Nanjing in 2022 and 2023 for phenotype comparison.

The leaves at V3 stage were collected for resequencing of soybean population (Supplementary Data Set 1). The seeds at 21 DAF were collected from 10:00 AM to 11:00 AM in August 2019 for 3′ RNA-seq (Supplementary Data Set 4). After collection, the tissues were immediately frozen in liquid nitrogen.

### DNA isolation and sequencing

Genomic DNA was isolated from leaves using the CTAB method (Allen et al. 2006). The genomic DNA was subjected to construct resequencing libraries using VAHTS Universal DNA Library Prep Kit for Illumina V4 (Vazyme, Jiangsu, China) according to the manufacturer's instructions. The libraries were sequenced on NovaSeq platform (Illumina) for 150 bp paired-end reads.

### RNA isolation and RT-qPCR

Total RNA was extracted using RNAiso Plus kit (Takara, Beijing, China) according to the manufacturer's instructions. After DNase treatment, reverse transcription was performed using PrimeScript RT reagent Kit with gDNA Eraser (Takara, Beijing, China). Then the cDNA was used as the template for qPCR which was performed using AceQ qPCR SYBR Green Master Mix (Vazyme, Jiangsu, China) on the CFX96 Touch Real-Time PCR Detection System (Bio-Rad, CA, USA) with three biological replicates. Gene expression level was normalized to the expression of *tubulin* gene (Glyma.03g124400) and calculated for each sample from the 2^−ΔΔCT^ method. The primers used in RT-qPCR are listed in Supplementary Table S2.

### Library construction and data analysis of 3′ RNA-seq

The 3′ RNA-seq libraries were constructed for 238 soybean accessions as described (Sanfilippo et al. 2017). Briefly, after ∼1 μg RNA was fragmented, oligo-dT index primers were used to synthesize the first-strand cDNA. After synthesis of double-stranded DNA, end repair, dA-tailing, adapter ligation, and PCR amplification were performed to generate 3′ RNA-seq libraries. The libraries were sequenced on NovaSeq platform (Illumina) for 150 bp paired-end reads.

Raw data were filtered to remove adapter sequences and low-quality bases by Fastp (version 0.21.0) (Chen et al. 2018). The clean data were mapped to the soybean reference genome (Gmax_508_a4.v1.0) using Hisat2 (version 2.1.0) (Kim et al. 2019). Only uniquely mapped reads were extracted for downstream analysis. The gene count matrix was generated using StringTie (version 1.3.3b) and the expression level [transcripts per million (TPM)] was calculated for each gene by edgeR (Pertea et al. 2015).

### Measurement of seed oil content

The oil content in soybean was measured as described (Liu et al. 2014). In brief, about 20 dry seeds from each accession were ground to a fine powder and 100 mg of seed powder was added to 500 μl of the prepared 95% isopropanol (v/v) followed by thorough mixing. After overnight rotation at 52 °C in an incubator and centrifuge, the isopropanol supernatant was transferred to a new centrifuge tube and the sediment was washed by isopropanol again. After the complete evaporation of the isopropanol, the oil content was calculated according to the weight change of the centrifuge tube divided by original weight of seed powder.

### Plasmid construction and transformation

To knock out *GmRWOS1* and *GmUSPL1*, two sgRNAs with low off-target scores (http://crispr.hzau.edu.cn/CRISPR2/) were used and directly synthesized with the GmU6 promoter as a cassette (Liu et al. 2017). The sgRNA cassette was constructed into CRISPR/Cas9 binary vector pCBSG015. The recombinant vectors were introduced into *Agrobacterium tumefaciens* strain EHA101 and used to transform soybean W82 by *A. tumefaciens*-mediated transformation. The transgenic plants were genotyped to identify deletion events near the targeted sites using PCR assay. Homozygous T2 transgenic lines were used for phenotypic analysis.

For overexpression of *GmRWOS1*, a 1,077 bp CDS sequence was amplified from soybean cultivar Williams 82 and ligated into the *BamHI/NcoI* sites of modified pFGC5941 vector. The construct was transformed into Williams 82 and the expression of *GmRWOS1* in transgenic plants were tested by RT-qPCR analysis. The T2 transgenic lines overexpressing *GmRWOS1* were used for phenotypic investigation. The related primers are listed in Supplementary Table S2.

### Variant calling and annotation

The resequencing data of the 421 soybean accessions were filtered using Fastp software (version 0.21.0) and then mapped to the reference genome (Gmax_508_a4.v1.0) by BWA program (0.7.17-r1188) with parameters (mem -M) (Li 2013; Chen et al. 2018). Duplicated reads were removed using Picard Toolkit (http://broadinstitute.github.io/picard/, v.2.18.15) and uniquely mapped paired reads were kept using SAMtools (-f 3 -q 10) for further analysis (Li et al. 2009). Variant calling was carried out by the Genome Analysis Toolkit (GATK, v.4.1.3.0) with the following parameters: –min-base-quality-score 25 && “QD < 2.0 || MQ < 40.0 || FS > 60.0 || SOR > 4.0 || MQRankSum < −12.5 || ReadPosRankSum < −8.0”–filter-name “Fail” for SNP filter && “QD < 2.0 || FS > 200.0 || SOR > 10.0 || InbreedingCoeff < −0.8 || ReadPosRankSum < −20.0”–filter-name “Fail” for INDEL filter (McKenna et al. 2010). Heterozygous variants were set as missing data if genotype quality (GQ) < 20 or sequencing depth (DP) < 4, and homozygous variants with DP < 2 were treated as missing data. Nucleic acid sites with heterozygote calls in more than 2% accessions were further discarded. Annotations of SNPs and INDELs were performed based on a gene model of the William82 genome using SnpEff (version 4.3t) (Cingolani et al. 2012).

### GWAS for seed weight and oil content

The phenotypic data including oil content and seed weight of 421 soybean accessions were collected in 2019 and 2020 for GWAS. A PCA of the population was conducted with independent SNPs (PLINK; MAF ≥ 0.05 & missing rate < 0.05 & –indep-pairwise 50 10 0.2) using GCTA software (version 1.92.3) with the following parameters: –make-grm –pca (Purcell et al. 2007; Yang et al. 2011). A MLM was applied for GWAS with a total of 1.1 million SNPs and INDELs by MLMA leaving-one-chromosome-out (LOCO) function in GCTA (Yang et al. 2011). The MLMA LOCO strategy was used to estimate a genetic relationship matrix (GRM) from SNPs except those on the chromosome where candidate SNPs were located. The GRM was added as a random effect to reduce the impact of population structure when performing association analysis based on MLM. The GEC software was employed to compute the effective number of SNPs (N), and a suggestive significance level (*P* = 6.24 × 10^−6^) was determined using the Bonferroni correction method (Li et al. 2012). To determine the lead SNPs and their LD proxies, clumping was performed by PLINK (version 1.9) with the following parameters: –clump –clump-p1 9 × 10^−7^ –clump-p2 0.01 –clump-r2 0.5 –clump-kb 500 (Purcell et al. 2007).

The genetic correlation between two seed traits was determined using the GREML function of GCTA software (Yang et al. 2011). The effect of a variant on each trait was expressed as a signed *t*-value (effect/standard error of effect) and the Pearson correlation coefficient was calculated in R (version 4.1.2).

### Identification of eQTLs

The genotype and gene expression of 238 soybean accessions were used to detect eQTLs. After discarding genes with low expression level (TPM < 5 in more than 95% of accessions) and low expression variance (less than 2-fold change between 95th and 5th percentile of expression values), a total of 25,089 genes were obtained for subsequent analyses. The expression of each gene was quantile normalized and used as a phenotype for GWAS. Using LOCO function in GCTA software, eQTL-gene associations were identified according to threshold *P* < 1 × 10^−4^ (Yang et al. 2011). We clumped all eQTL-gene associations using an LD-threshold (r2) of 0.2 in a 250 kb radius by PLINK. The lead SNPs (*P* < 7.7 × 10^−7^; 1/*n*, *n* represents the total number of SNPs used in eQTL detection) with more than two LD proxies were retained as putative eQTLs (Purcell et al. 2007). The putative eQTLs which were identified in a LD block via PLINK (−blocks no-pheno-req no-small-max-span –blocks-max-kb 5000 –blocks-strong-lowci 0.70 –blocks-strong-highci 0.98 –blocks-recomb-highci 0.9 –blocks-inform-frac 0.8) were further merged and represented by the most significant lead SNP (Purcell et al. 2007). According to the distance (cutoff: 1 Mb) between lead SNPs and associated genes, the eQTLs were classified as local or distal eQTLs in subsequent analysis. Using a 1 Mb sliding window with a 100 kb step, distal eQTL hotspots were detected based on the mapped gene numbers greater than the threshold (15; *P* < 0.01) which was calculated from the distribution of the maximum number of mapped genes in 1,000 random permutations with shuffling positions of distal eQTLs.

### Construction of coexpression module

To identify modules of coexpression genes, the centered and standardized expression matrices, in which rows represent genes and columns represent individuals, were used as input for ICA to deconvolve factor loadings (module eigengene profile) for each individual and gene-level weights of the factor. The fastICA algorithm was performed by runICA function from R package picaplot (version 0.99.7) with the following parameters: var_cutoff = 70, max_iter = 10, *n*_runs = 15, *n*_cores = 5 (https://github.com/jinhyunju/picaplot). Pairwise spearman correlation coefficients of genes in module IC79 were calculated as edge information to construct coexpression network. The module IC79 was partitioned into three submodules based on gene expression connectivity via louvain algorithm in python-igraph (version 0.8.2) (https://igraph.org/). The network graph was visualized by Gephi software (version 0.10.1) (Bastian et al. 2009). The relevant genes involved in oil metabolism were obtained from *ARALIP* website (http://aralip.plantbiology.msu.edu/pathways/pathways).

### Identification of distal eQTLs for coexpression modules

Using module expression as a phenotype, we performed GWAS to identify 30 genomic loci via LOCO function in GCTA software (Yang et al. 2011). Considering that local eQTLs of genes within modules could also be detected through GWAS analysis of modules, we further distinguish true distal eQTLs from local eQTLs for coexpression modules. Based on 1,357 genes in the module IC79, 1,000 permutations of 1,357 randomly selected genes from the genome were conducted to construct a stochastic distribution of the maximum number of distal genes regulated by the 30 genomic loci. The distal eQTL of a module was defined as the number of genes in a module regulated by the locus exceeding the threshold 14 (*P* < 0.01). To identify the candidate TFs in the distal eQTL of a module, promoter sequences (2 kb) of all genes in the module were extracted for motif enrichment analysis by SEA function in MEME Suite (Bailey et al. 2009).

### TWAS for seed weight and oil content

To perform TWAS, genes exceeding the heritability threshold (GCTA) were retained to calculate expression weights based on the relationship between expression levels and variants in the 500 kb flanking regions of genes via FUSION software with parameters (−models top1, blup, bslmm, lasso, enet) (Gusev et al. 2016). According to *P*-value and effect size of GWAS, we computed Z scores and conducted TWAS using FUSION (Gusev et al. 2016). Candidate genes were determined based on a threshold of |3| of Z-score in TWAS and eGWAS for both seed weight and oil content.

### Genetic diversity analysis

The SNP data of 2,898 soybean accessions were downloaded from the Genome Variation Map database in BIG Data Center (GVM000063). To transfer coordinates of the SNPs from soybean reference genome of ZH13 to William82, we performed genome alignment by Minimap2 (version 2.24-r1122) with the following parameters: -cx asm5 –cs. We created a chain file by transanno (version 0.3.0; https://github.com/informationsea/transanno). Then, the coordinates of the SNPs were updated by CrossMap (version 0.6.4) (Zhao et al. 2014; Li 2018). Using a 100 kb window and a 10 kb step, π values were calculated via VCFtools (version 0.1.16), and selective sweeps were defined as regions with the top 5% of π ratios (wild vs landrace and landrace vs cultivar) (Danecek et al. 2011). For genetic diversity of *GmRWOS1*, π and *F*_ST_ values were calculated with 20 kb window and 5 kb step using VCFtools (Danecek et al. 2011). The genome-wide threshold was defined as the top 10%.

### Accession numbers

The genome resequencing data of 421 soybean accessions and 3′ RNA-Seq data of 238 soybean accessions are available in Genome Sequence Archive (GSA) database in the National Genomics Data Center (https://bigd.big.ac.cn/gsa/) under accession number PRJCA016545.

## Supplementary Material

koae062_Supplementary_Data

## Supplementary data

The following materials are available in the online version of this article.

**Supplementary Figure S1.** Phenotype divergence among accessions in different regions.

**Supplementary Figure S2.** Characteristics of transcriptome data of seeds at 21 DAF from 238 accessions.

**Supplementary Figure S3.** Distribution of local eQTLs with different distances to transcription start site (TSS) of regulated eGenes.

**Supplementary Figure S4.** GO and KEGG terms enriched in the eGenes regulated by eQTL hotspots.

**Supplementary Figure S5.** Correlation with seed traits in other seed development stages.

**Supplementary Figure S6.** Verification of GmUSPL1 controlling seed traits.

**Supplementary Figure S7.** The difference of genes regulated by two eQTL hotspots and other genes in module IC79.

**Supplementary Figure S8.** Haplotype analysis of *MYC2*, *GmBZR1* and *bZIP44*.

**Supplementary Figure S9.** Module IC110 involved in regulation of seed weight and oil content.

**Supplementary Figure S10.** The relationship between seed trait and TWAS candidate gene aggregation in independent soybean population.

**Supplementary Figure S11.** Verification of GmRWOS1 controlling seed traits.

**Supplementary Table S1.** GWAS loci for module IC79.

**Supplementary Table S2.** Primers used in this study.

**Supplementary Data Set S1.** Sample information.

**Supplementary Data Set S2.** Mapping statistics of resequencing.

**Supplementary Data Set S3.** GWAS significant loci.

**Supplementary Data Set S4.** Mapping statistics of 3′ RNA-seq.

**Supplementary Data Set S5.** eQTL-eGene list.

**Supplementary Data Set S6.** Genes related to storage proteins, oil metabolism, and seed weight.

**Supplementary Data Set S7.** Genes in module IC110.

**Supplementary Data Set S8.** Genes in module IC79.

**Supplementary Data Set S9.** Statistical analyses in this study.

## Dive Curated Terms

The following phenotypic, genotypic, and functional terms are of significance to the work described in this paper:

BZR1 Gramene: AT1G75080
BZR1 Araport: AT1G75080

DA1 Gramene: AT1G19270
DA1 Araport: AT1G19270

PMI1 Gramene: AT1G42550
PMI1 Araport: AT1G42550

ARF2 Gramene: Mp4g11820.1
ARF2 Araport: Mp4g11820.1

BCCP2 Gramene: AT5G15530
BCCP2 Araport: AT5G15530
