# Supplementary material for: Integrative omics analysis elucidates the genetic basis underlying seed weight and oil content in soybean
Source: Plant Cell. 2024 Feb 27;36(6):2160–75. doi: 10.1093/plcell/koae062 (PMC11132872; doi:10.1093/plcell/koae062)
Supplement: koae062_Supplementary_Data [file koae062_supplementary_data.zip › tpc.23.01260Supplemental Figures and Tables.pdf]

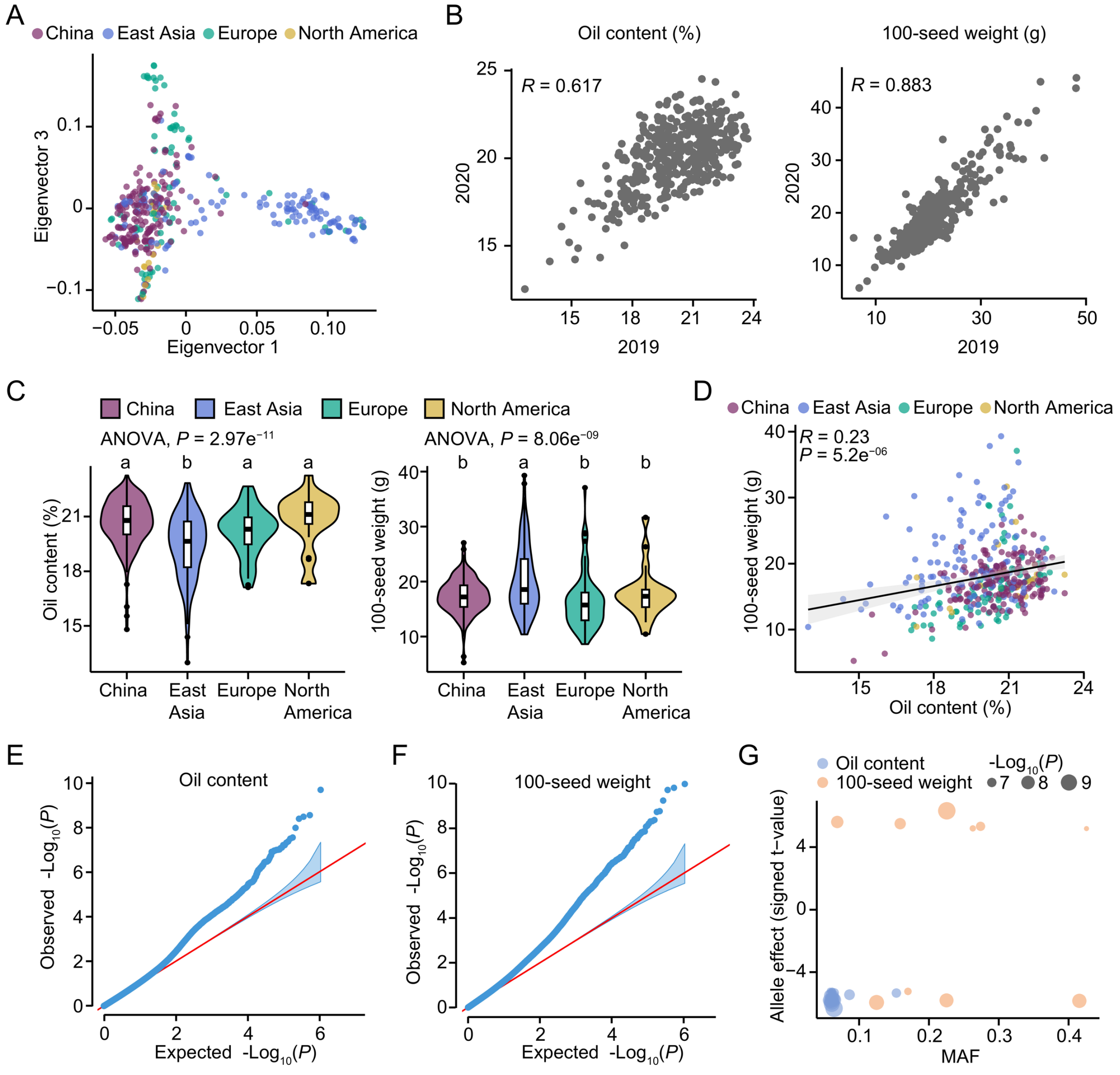

Supplemental Figure S1. Phenotype divergence among accessions in different regions. (Supports Figure 1)

(A) Principal Component Analysis (PCA) of the first and third principal components for 421 soybean accessions. **(B)** The scatter plots of oil content and 100-seed weight in 2019 and 2020 at Harbin. **(C)** The distributions of seed oil content (left) and 100-seed weight (right) of accessions in China (n = 187), East Asia (n = 130), Europe (n = 83) and North America (n = 17), respectively. In each box plot, borders represent the first and third quartiles, center line denotes median, and whiskers extend to 1.5 times the interquartile range beyond the quartiles. Different letters indicate statistically significant differences between groups determined using ANOVA and Tukey's honestly significant difference test ($P < 0.05$). **(D)** The correlation between oil content and 100-seed weight. **(E, F)** Quantile-quantile plot for oil content (**E**) and 100-seed weight (**F**). **(G)** The relationship between minor allele frequency (MAF) and effect size (signed t-value) of significant variant ($P < 6.24 \times 10^{-6}$) for oil content and 100-seed weight based on published population.

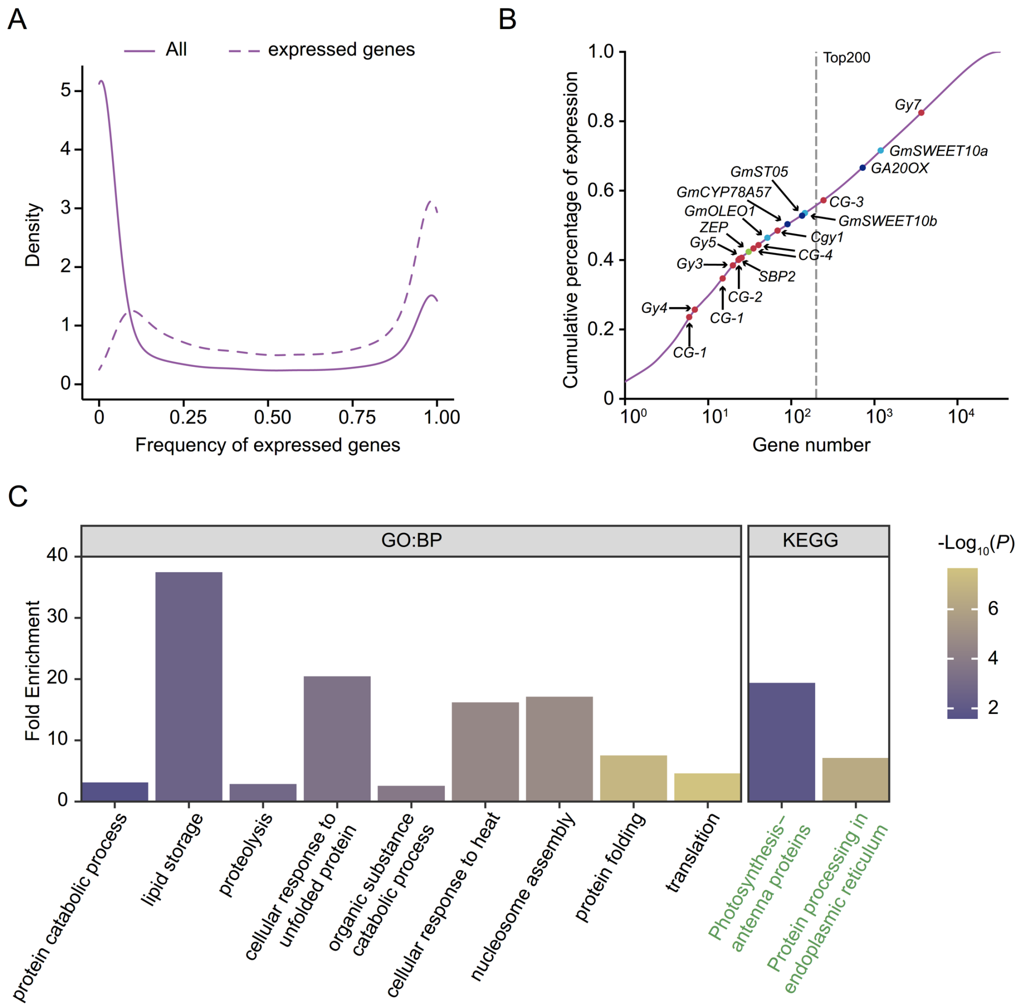

Supplemental Figure S2. Characteristics of transcriptome data of seeds at 21 DAF from 238 accessions. (Supports Figure 2)
(A) Frequency distribution of expressed genes in 238 individuals. The solid line indicates all genes for statistics, and dashed line represents genes expressed in more than 5% of individuals. **(B)** Cumulative distribution of fraction of total transcriptions contributed by genes sorted from highest to lowest expression. The dashed line indicates the cutoff of top 200 most expressed genes. **(C)** GO and KEGG enrichment terms of the top 200 highest expressed genes. The GO and KEGG terms are labeled in black and green font, respectively.

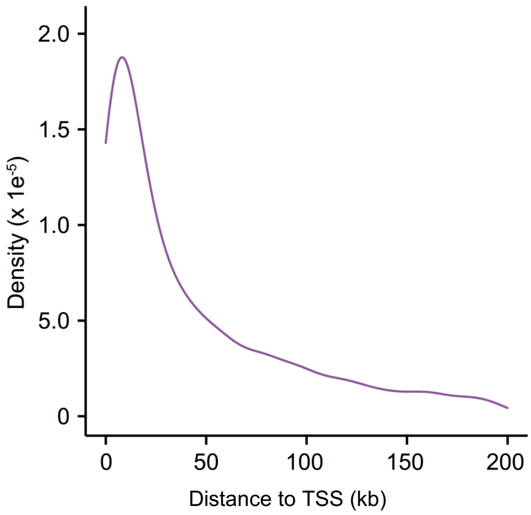

Supplemental Figure S3. Distribution of local eQTLs with different distances to transcription start site (TSS) of regulated eGenes. (Supports Figure 2)

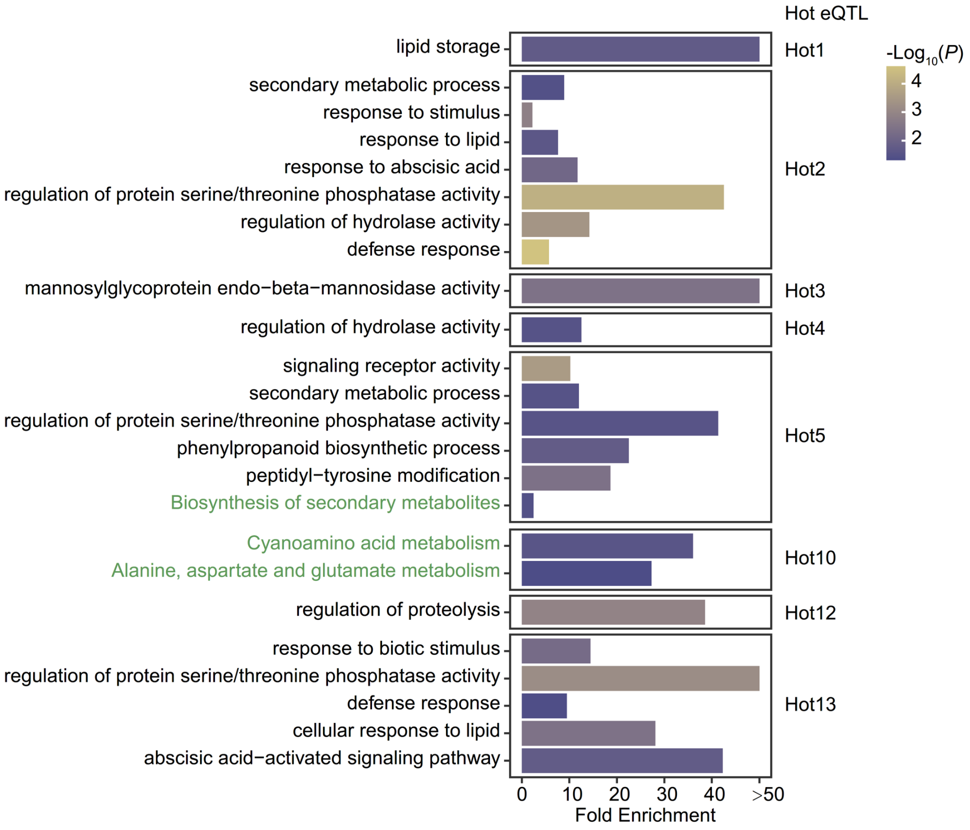

Supplemental Figure S4. GO and KEGG terms enriched in the eGenes regulated by eQTL hotspots. (Supports Figure 2)
The GO and KEGG terms are labeled in black and green font, respectively.

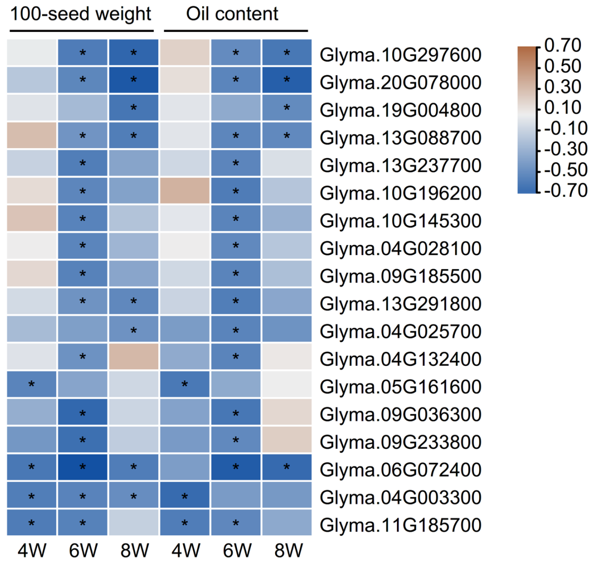

Supplemental Figure S5. Correlation between seed traits and gene expression in seeds at different development stages. (Supports Figure 4)
Heatmap of correlation between seed traits and gene expression in seeds at 4, 6 and 8 weeks after flowering using 26 soybean accessions. Asterisks indicate significant correlation (*P* < 0.01, Pearson correlation)

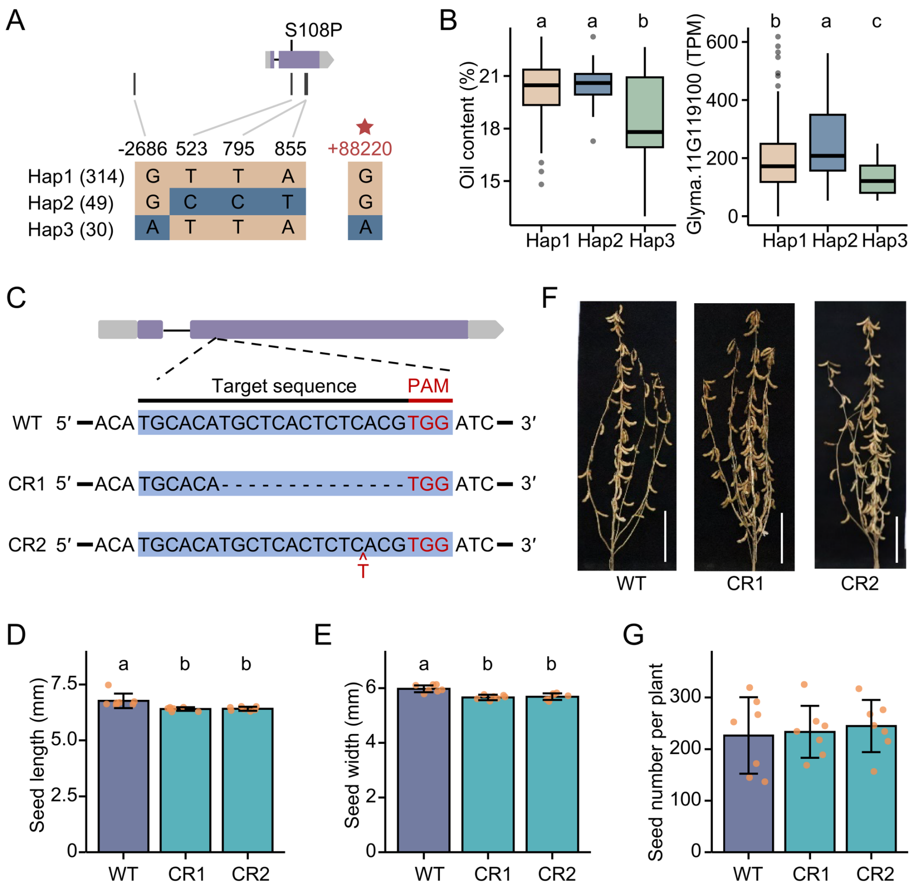

Supplemental Figure S6. Verification of *GmUSPL1* controlling seed traits. (Supports Figure 4)

(**A**) The spectrum of three haplotypes for *Glyma.11G119100*. Vertical lines represent variants. The lead SNP for oil content is marked in red star. **(B)** Differences of oil content (left) and *Glyma.11G119100* expression (right) between individuals with different haplotypes (Hap1, n = 314; Hap2, n = 49; Hap3, n = 30). A one-way ANOVA and SNK test were used for statistical analysis. In each box plot, borders represent the first and third quartiles, center line denotes median, and whiskers extend to 1.5 times the interquartile range beyond the quartiles. Different letters indicate statistically significant differences between groups determined using ANOVA and Tukey's honestly significant difference test ($P < 0.05$). **(C)** Schematic diagram of the gene structure of *GmUSPL1* and the sequence changes in two *gmuspl1* mutants (CR1 and CR2) generated by the CRISPR/Cas9 system. **(D, E)** Seed length and seed width in wild type, CR1 and CR2 mutants. **(F)** Architecture of wild type, CR1 and CR2 mutants at the harvest stage. Scale bar, 18 cm. **(G)** Seed number per plant in wild type, CR1 and CR2 mutant. Data in (D, E, G) are mean ± SD (n = 7). Two-tailed t-test was used for statistical analysis (CR1 or CR2 compared to WT, $P < 0.05$). The value of each plant is represented by a dot.

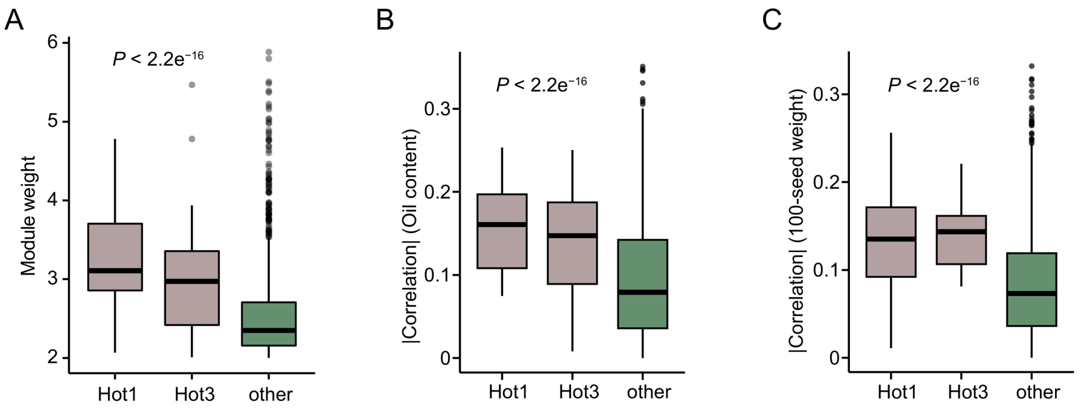

Supplemental Figure S7. The difference of genes regulated by two eQTL hotspots and other genes in module IC79. (Supports Figure 4)

(A-C) Module weight (**A**), correlation between oil content and expression (**B**) and correlation between 100-seed weight and expression (**C**) of genes regulated by Hot1 and Hot3 compared with other genes in module IC79 (Hot1, n = 33; Hot3, n = 13; other, n = 1,311). In each box plot, borders represent the first and third quartiles, center line denotes median, and whiskers extend to 1.5 times the interquartile range beyond quartiles. Two-sided Wilcoxon rank sum test was used for statistical analysis (Hot1 or Hot2 compared to other).

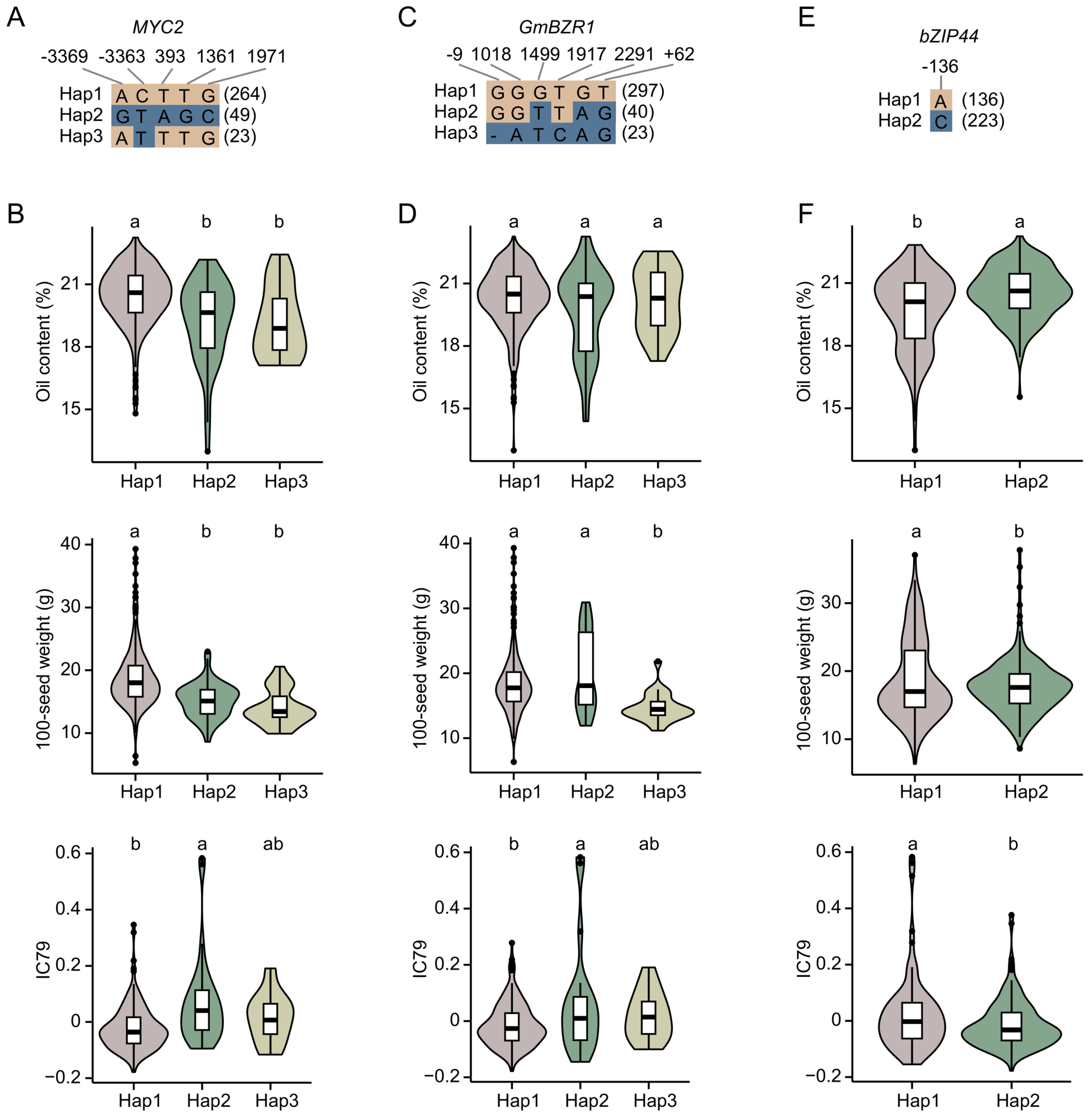

Supplemental Figure S8. Haplotype analysis of *MYC2*, *GmBZR1* and *bZIP44*. (Supports Figure 4)

(A) Haplotypes of *MYC2*. **(B)** Differences of oil content, 100-seed weight and IC79 expression among individuals with the different haplotypes of *MYC2* (Hap1, n = 264; Hap2, n = 49; Hap3, n = 23). **(C)** Haplotypes of *GmBZR1*. **(D)** Differences of oil content, 100-seed weight and IC79 expression among individuals with the different haplotypes of *GmBZR1* (Hap1, n = 297; Hap2, n = 40; Hap3, n = 23). **(E)** Haplotypes of *bZIP44*. **(F)** Differences of oil content, 100-seed weight and IC79 expression among individuals with the different haplotypes of *bZIP44* (Hap1, n = 136; Hap2, n = 223). In each box plot, borders represent the first and third quartiles, center line denotes median, and whiskers extend to 1.5 times the interquartile range beyond the quartiles. Different letters indicate statistically significant differences between groups determined using ANOVA and Tukey's honestly significant difference test ($P < 0.05$).

Supplemental Data. Yuan et al. (2024). Co-regulation of seed oil and weight. Plant Cell.

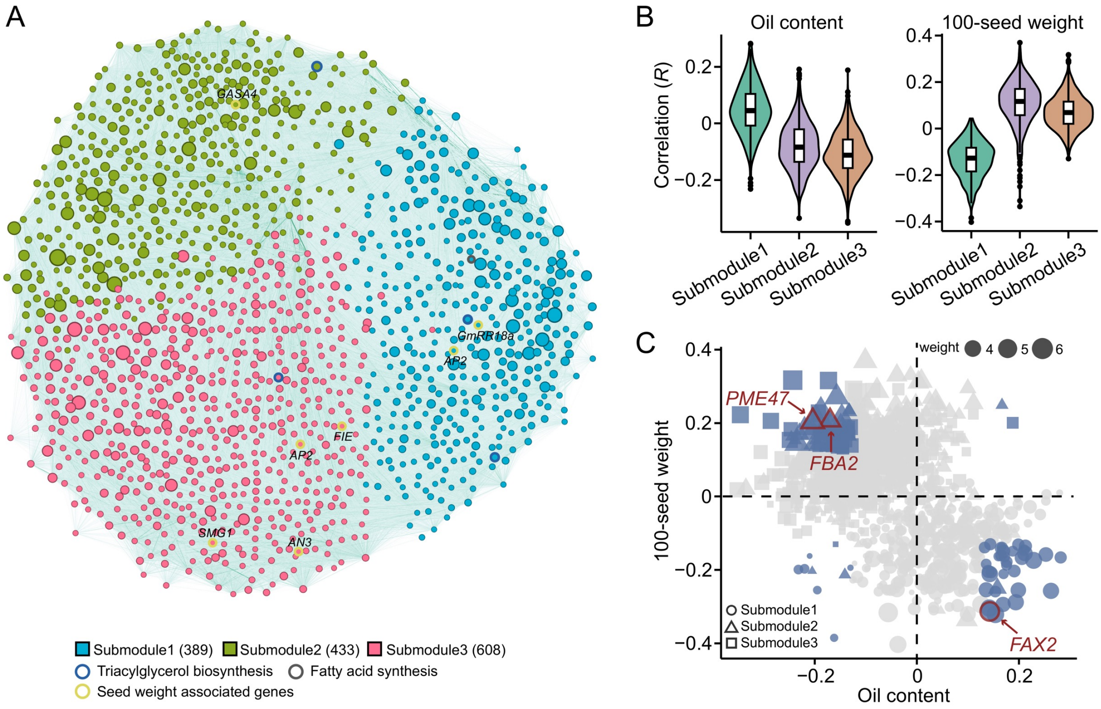

Supplemental Figure S9. Module IC110 involved in regulation of seed weight and oil content. (Supports Figure 3)

(A) Correlation network of genes in module IC110. The genes related to seed weight and lipid biosynthesis are labeled in the network. **(B)** Correlation of genes in different submodules (Submodule1, n = 389; Submodule2, n = 433; Submodule3, n = 608) with 100-seed weight (right) and oil content (left). In each box plot, borders represent the first and third quartiles, center line denotes median, and whiskers extend to 1.5 times the interquartile range beyond the quartiles. **(C)** The scatter plot of correlation of genes in three submodules with 100-seed weight (y-axis) and oil content (x-axis). The genes significantly associated with both 100-seed weight and oil content are marked in blue (*P* < 0.01, Pearson correlation).

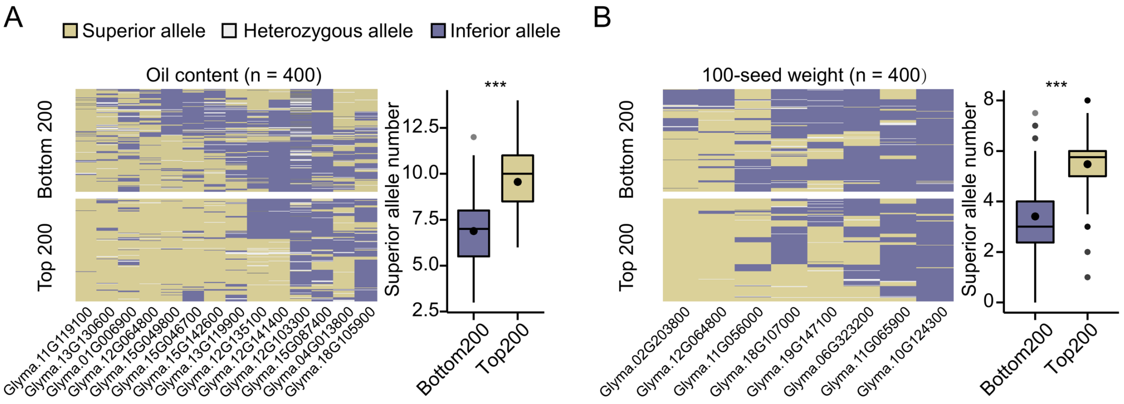

Supplemental Figure S10. The aggregation of superior alleles in another independent soybean population. (Supports Table 1)

(A, B) The aggregation of superior alleles in the top and bottom 200 accessions with highest and lowest oil content (**A**) or 100-seed weight (**B**) in previously re-sequenced population, respectively (n = 200). The gray squares in heatmaps represent missing data. In each box plot, borders represent the first and third quartiles, center line denotes median, and whiskers extend to 1.5 times the interquartile range beyond the quartiles. Two-sided Wilcoxon rank sum test was used for statistical analysis. Significance was denoted by three asterisks (***), indicating $P < 0.001$.

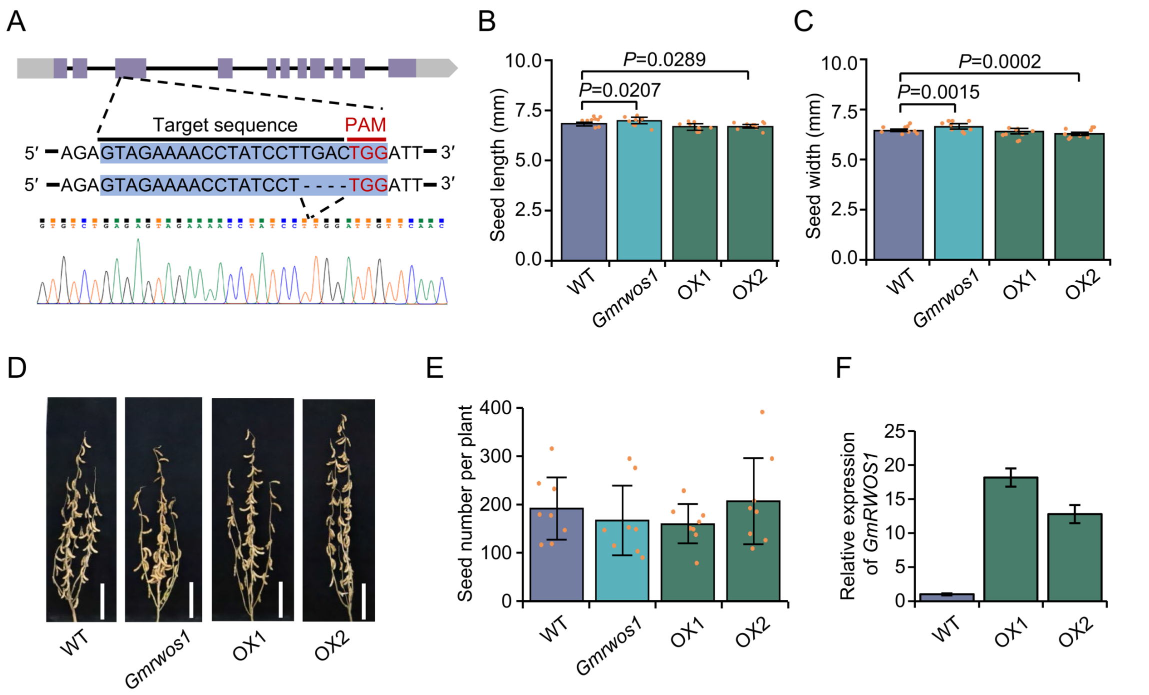

Supplemental Figure S11. Verification of *GmRWOS1* controlling seed traits. (Supports Figure 6)

(A) Schematic diagram of the gene structure of *GmRWOS1* and the sequence change in *gmrwos1* mutant induced by the CRISPR/Cas9 system. **(B, C)** Seed length and seed width in wild type, *gmrwos1* mutant and overexpression transgenic lines OX1/2. Data are mean ± SD (n = 9). Two-tailed t-tests were used for statistical analysis. The value of each plant is represented by a dot. **(D)** Architecture of wild type, *gmrwos1* mutant and overexpression transgenic lines OX1/2 at the harvest stage. Scale bar, 18 cm. **(E)** Seed number per plant in wild type, *gmrwos1* mutant and overexpression transgenic lines OX1/2. Data are mean ± SD (n = 8). Two-tailed t-tests were used for statistical analysis. The value of each plant is represented by a dot. **(F)** Relative expression of *GmRWOS1* in the leaves of two overexpression transgenic lines OX1/2 compared with wild type. Data are mean ± SD (n = 3).

Supplemental Table S1. GWAS loci for module IC79

Chromosome	SNP	P value	Range of GWAS locus
2	Gm02:50045782	1.09E-07	Gm02:49941256..50170542
4	Gm04:48786487	3.28E-08	Gm04:48618989..48786487
4	Gm04:6550025	1.59E-11	Gm04:6372438..7027530
8	Gm08:17557439	4.84E-08	Gm08:17127724..17789271
8	Gm08:35072873	5.91E-07	Gm08:34593708..35175509
12	Gm12:34433019	7.65E-09	Gm12:34020759..34650266
12	Gm12:7976271	8.93E-08	Gm12:7738793..8402252
14	Gm14:20612686	1.57E-08	Gm14:20148508..27982719
16	Gm16:32998631	8.04E-08	Gm16:32328834..33431367
17	Gm17:40725894	1.20E-09	Gm17:40336436..40917693
18	Gm18:10372339	2.57E-08	Gm18:10006010..10643271
18	Gm18:56229040	6.08E-09	Gm18:56195362..56443166
18	Gm18:882374	1.10E-08	Gm18:881311..896419
19	Gm19:6683423	5.26E-07	Gm19:6683423..7173482

Supplemental Table S2. Primers used in this study

Primer	Sequence	Purpose
GmRWOS1-clon-F	ATGTCCGAACCTCATTCATCG	Gene cloning
GmRWOS1-clon-R	CTATTGTGATTTTGGCTTGGCAATC	
GmRWOS1-F	AAGATGGCATTTCTCCCGCA	RT-qPCR
GmRWOS1-R	GGCTCCAAAGTTGAACAATCCA	
Tubulin-F	GGAGTTCACAGAGGCAGAG	RT-qPCR
Tubulin-R	CACTTACGCATCACATAGC	
GmRWOS1-F1	TCCTAGAAGGGGGAGGATGT	Confirmation of mutation
GmRWOS1-R1	ACCAGTGACGAAAGACCATACA	
GmUSPL1-F1	ATCACGGTACATTTAATGCCCA	Confirmation of mutation
GmUSPL1-R1	ATCTTGGAAGCTGGGATTTCTATG	
