# Supplementary material for: Integrative omics analysis elucidates the genetic basis underlying seed weight and oil content in soybean
Source: Plant Cell. 2024 Feb 27;36(6):2160–75. doi: 10.1093/plcell/koae062 (PMC11132872; doi:10.1093/plcell/koae062)
Supplement: koae062_Supplementary_Data [file tpc.01260.2023-s05.pdf › tpc.01260.2023-s05.pdf]

THE PLANT CELL

Integrative omics analysis elucidates the genetic basis underlying regulation of seed weight and oil content in soybean

Xiaobo Yuan, Xinyu Jiang, Mengzhu Zhang, Longfei Wang, Wu Jiao, Huatao Chen, Junrong Mao, Wenxue Ye, and Qingxin Song

Review Timeline:

Submission Date:	16-Jun-2023
Editorial Decision:	30-Jul-2023
Resubmission Received:	03-Jan-2024
Editorial Decision:	20-Jan-2024
Revision Received:	29-Jan-2024
Accepted:	03-Feb-2024

Dr. Qingxin Song
Nanjing Agricultural University
Nanjing, Jiangsu 210095
China

Dear Dr. Song:

Thank you for choosing to send your manuscript entitled "Integrative omics analysis elucidates the genetic basis underlying synergistic regulation of seed weight and oil content in soybean" for consideration at The Plant Cell. Your submission has been evaluated by members of the editorial board as well as expert reviewers in your field, and we regret to inform you that we are not able to recommend the publication of this manuscript in its current form. We have had input from multiple scientists and we have solicited post-review comments.

The method of integrated analyses (GWAS and TWAS) as well as the cut-off used need to be re-evaluated. The validation of two candidate genes needs additional data to support the conclusions that you made. In additional, how the RWOS1 gene negatively affects seed weight and oil content in the same direction needs additional verification.

That said, we would be willing to re-consider a new manuscript that fully addressed the concerns raised during this review process. If you decide to resubmit to The Plant Cell, it will be evaluated as a new submission subject to full assessment by the editorial board, and if sent for external review, a new set of reviewers might be chosen. Reviewers will be asked to assess your work as a new manuscript, and not only whether previous reviewer comments have been addressed.

In addition, we find that the writing in general lacks clarity and conciseness. We recommend the use of a professional science editing service, not merely for language/grammar editing, but to aid in reorganizing as well as rewriting the text to clarify the background, purpose, and flow of the study. Several editing services have agreed to extend a discount to authors coming to their website via this ASPB page https://aspb.org/aspb-journal-submission-editing-services.

It will be important to convince the editors and reviewers that the study adds significant new understanding(s) of mechanisms or processes, or otherwise represents a significant advance in the field, and that the major claims made are fully justified by the data presented. This includes careful consideration and explanation of the various controls used in experiments, the extent and manner of replication, and the statistical analyses used. Sampling methods and the nature of "biological replicates" should be described precisely (i.e. different plants, parts of plants, pooled tissue, independent pools of tissue, sampled at different times, etc.), along with a clear description of and rationale for any statistical analyses conducted. The reader should know exactly what was sampled; what forms the basis of the calculation of any means and other statistical variables and parameters reported. This is also necessary to ensure that proper statistical analysis was conducted.

The Plant Cell now requires authors to complete and submit an author revisions checklist upon submission of a revised manuscript. The aim of the checklist is to aid authors in preparing a high-quality manuscript, facilitate the review and assessment of revised manuscripts, and help to ensure that journal standards are maintained across the board. If your manuscript is accepted, the completed checklist will be published as supplemental material attached to the article online. Please download a copy of the checklist (a fillable PDF form) at this link, for submission with your revised manuscript: https://tpc.msubmit.net/html/Author_Revisions_Checklist.pdf.

Note also that supplemental materials should be restricted to large datasets and tables, presentation of replicates, and validation of reagents, methods, or genotypes. Any data that are used to support the main claims must be in the main manuscript. Supplemental figure legends must indicate what figure in the main manuscript is supported by the supplemental data presented.

We thank you for your interest in and support of The Plant Cell. We hope we will be able to render a more positive decision on future work.

On behalf of the editorial board,

Reviewing Editor, Board of Reviewing Editors
James Birchler, Senior Editor
Blake Meyers, Editor-in-Chief

The Plant Cell

--- Reviewer comments:

Reviewer #1 (Comments for the Author):

The study conducted large-scale GWAS and TWAS using 421 soybean accessions to investigate potential genetic loci, regulatory modules, and candidate genes that regulate seed traits. The authors also performed functional analyses of two genes through knockout and/or overexpression in plants. Although the study may provide valuable data for further understanding the regulatory mechanisms of seed weight and oil content in soybean, the main findings and conclusions drawn from these large-scale data seem unclear. The manuscript primarily presents descriptive results, and the true value of this information for subsequent studies needs to be tested.

1. The study does not convincingly reveal the genetic basis for synergistic regulation of seed weight and oil content as suggested by the title. The authors' analysis treats whole seeds as one tissue, without the development stage analysis, and it remains unclear how seed weight and oil content are synergistically regulated based on the findings presented in the manuscript.

2. The authors acknowledge the existence of numerous GWAS studies on seed traits in soybean, and the major challenge lies in identifying specific genes underlying these traits. Although the study identifies some candidate genes and attempts to verify their functions, the significance of the GWAS-TWAS approach used here is not sufficiently validated. The USPL1 was selected due to its homology to the Arabidopsis, whereas the RWOS1 tends to play quite a minor role in regulating seed traits according to the authors' data. Besides, even they regulate seed traits, natural variations and their contributions to seed traits require further examination.

3. The selection analysis of RWOS1 might benefit from a whole-genome scale evaluation rather than being limited to the local region, despite this kind of analyses has been widely published in the literature.

Reviewer #2 (Comments for the Author):

Seed weight and oil content are two most important determinants of soybean yield and quality. There is a high correlation between seed weight and oil content. Hundreds of quantitative trait loci (QTLs) controlling seed weight and oil content have been identified, but the genetic mechanism and causal genes underlying QTLs synergistically regulating seed weight and oil content remain largely unknown. Here, the authors explored genetic loci and identified co-expression modules and candidate genes that significantly associated with seed weight and oil content in soybean by integration of GWAS, expression quantitative trait locus (eQTL) information and TWAS. Candidate causal genes GmRWOS1 and GmUSPL1 were further studied using genome-editing strategy and resulted in synergistical variation in seed weight and oil content. This is an interesting and important work and with clearly presented results that should be of wide interest to Large-Scale Biology community and those interested in breeding of high-yield and high-quality varieties in soybean. I recommend acceptance following satisfactory revision. However, some concerns need to be addressed.

Major comments:
1. How to control false positive signals caused by population structure when performing GWAS? I did not find this in the METHOD.
2. Are there any correlations between the phenotype data from two different years? Additionally, what is the level of correlation between seed weight and oil content?
3. How were the 238 accessions selected for eQTL analysis? Do they have the same population composition as the 421 accessions? Was the issue of false positive signals caused by population structure considered when performing eGWAS?
4. Is there always a positive relationship between seed weight and oil content? It would be helpful to clarify this in the introduction section (page 3:64-67).
5. In the case of eQTL hotspots, they may regulate multiple genes. However, it's also possible that an "eQTL hotspot" only regulates one upstream gene, such as a transcription factor. In such cases, downstream genes may exhibit similar expression patterns to that of the upstream gene. During eGWAS, these downstream genes might be considered as direct targets of the eQTL hotspot by mistake. How did the authors distinguish between these two situations?
6. I am a little bit curious about that the growth condition of GmRWOS1 knockout lines and overexpression lines? I couldn't find this in METHOD. I would also like to see some discussion on GmRWOS1 negatively regulates seed weight and oil content in soybean.
Minor comment:
1. Please clarify the line color used in the FST figure.

Reviewer #3 (Comments for the Author):

In this manuscript, Yuan et al dissected the genetic architectures of seed weight and oil content by combining GWAS and TWAS

using 421 soybean accessions. They also performed eQTL analysis using the transcriptional data of 238 soybean accessions. The integration of multiple-omics analyses identified and validated GmRWOS1 and GmUSPL1 to be involved in regulation of seed weight and oil content in soybean. It is a very interesting work and the manuscript is well written, which also provides a good resource for soybean functional study, particularly for seed development. However, some points are not clear in the current version, which may need to be further clarified or investigated.

1. The authors collected the soybean accessions from 24 countries in four main regions with similar latitude. Is this true? Based on the map, looks like the accession ranged a lot in the latitudes. Please confirm.
Then, the author phenotype them in three locations, Harbin, Nanjing, Sanya. If the accessions from similar latitude, I am not sure if this kind of design using phenotype from three locations are good or not. As soybean is quite sensitive to environment, I believe the accessions may not grow normally in either two locations of Harbin, Nanjing, or Sanya as they are from quite different latitude, since the soybeans mainly come from the similar latitude. If the accessions from similar latitude, I prefer using data from the same location from different years, which may be more reasonable.
The authors need to provide the phenotypic divergence among different locations, and also to check the consistence among GWAS results using individual environment data.

2. It is very interesting for the phenotyping results, the authors found North America exhibited the highest oil content, while accessions from East Asia showed the lowest oil content, but highest 100-seed weight. As the authors cited and found, the seed weight and oil content usually showed positive correlation. Why this is not the case for that from the East Asia? Are the accessions showed highest 100-seed weight same to that showed lowest oil content? This may need to be checked and to give a description. If this is the case, it means that some genes are specifically broke the co-regulation of seed weight and oil content in the East Asia soybean, which is more interesting that deserve to be deeply investigated.

3. Using GWAS, a total of 21 and 13 significantly associated loci were detected for oil content and 100-seed weight, respectively. Following, the authors using TWAS also identified some gene associated with oil content and 100-seed weight, respectively. What is overlap of these two dataset? Are the TWAS genes from individual GWAS regions, or may some come from the same GWAS regions? This data need to be provided.

4. The authors found significant positive genetic correlation between seed weight and oil content, then also found the minor alleles that exceeded significant threshold mainly showed negative effect for oil content, whereas most minor alleles exerted positive influence to seed weight. The results is very interesting. How to explain that the minor alleles showed negative effect for oil content but exerted positive influence to seed weight? Is there any specific cases to show these effects? It will be very interesting to give some examples to show this kind of genes may broke the co-regulation of seed weight and oil content in soybean.

5. For the eQTLs hotspots, I am a little bit confuse, or not clear. The authors found most of these hotspots overlapped with previously reported QTLs and known genes related to seed traits, especially seed weight or oil content. But GO and KEGG enrichment analysis revealed eGenes regulated by distinct eQTL hotspots were enriched in different terms. If most of these hotspots overlapped with previously reported QTLs and known genes related to seed traits, especially seed weight or oil content, the genes regulated by these eQTL hotspots should enriched some terms also related to seed weight or oil content, at least some are similar. However, almost no overlap for different eQTLs hotspots.
In addition, are the eQTLs all distal eQTLs? Or what is ratio?

6. for figure 3A, I am not quite sure why talk about protein, the whole story did not talk about protein.
In addition, why the top panel only shows 10 eQTLs hotspots, other eQTLs hotspots do not have these kind of pattern/relationship? Or this kind of relationship only randomly associated but did not provide too much clear clues?

7. Still Figure 3A, the authors found that two modules (IC79 and IC110) showed significant correlation with both 100-seed weight and oil content, whereas only IC79 (1,357 genes) exhibited identical direction of associations with both traits. I am more interesting in IC110, are the genes in this module only control oil content? Some specific cases?

8. The author found that Glyma.11G119100 showed positive correlation with both seed traits and was colocalized with loci significantly associated with oil content in GWAS as well. What is the causal genetic variation for this gene, which needs to be provided? In addition the authors only provided knock out line. Strictly, at least OE lines are also needed.

9. For Supplemental Figure S7, Haplotype analysis of MYC2, GmBZR1 and bZIP44, the expression did not show significant differences as the phenotype, which is not so consistent with the claim of the correlation between expression differentiation and phenotype changes.

Reviewer #1 (Comments for the Author):

The study conducted large-scale GWAS and TWAS using 421 soybean accessions to investigate potential genetic loci, regulatory modules, and candidate genes that regulate seed traits. The authors also performed functional analyses of two genes through knockout and/or overexpression in plants. Although the study may provide valuable data for further understanding the regulatory mechanisms of seed weight and oil content in soybean, the main findings and conclusions drawn from these large-scale data seem unclear. The manuscript primarily presents descriptive results, and the true value of this information for subsequent studies needs to be tested.

Response: We appreciate the encouraging and constructive comments from this expert reviewer and have addressed the concerns in this revision.

1. The study does not convincingly reveal the genetic basis for synergistic regulation of seed weight and oil content as suggested by the title. The authors' analysis treats whole seeds as one tissue, without the development stage analysis, and it remains unclear how seed weight and oil content are synergistically regulated based on the findings presented in the manuscript.

Response: Many thanks for this valid comment. We found 69 genes showing significant correlation with both 100-seed weight and oil content at 21 DAF in module IC79 in initial submission (Figure 4A). To further explore the genetic basis for synergistic regulation of seed weight and oil content, we analyzed the correlation between these genes and seed traits at different seed developmental stages (4, 6, 8 weeks after flowering) using published datasets (Liu et al., 2020). We found expression levels of 18 genes were significantly correlated with both seed weight and oil content in soybean seeds at different developmental stages (Supplemental Figure S5), such as *Glyma.19G004800* for oleosin synthesis and *Glyma.10G297600* for lipid transport. These results suggest coordinated expression of these candidate genes at different developmental stages may mediate synergistic regulation of seed weight and oil content.

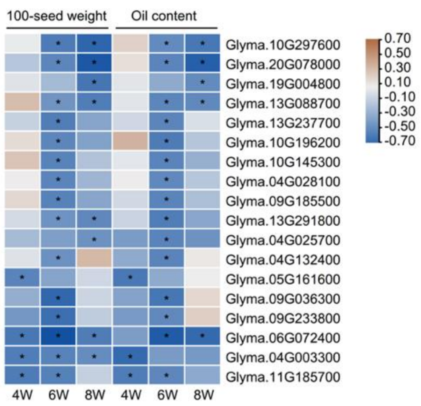

Supplemental Figure S5. Heatmap of correlation between seed traits and gene expression in seeds at 4, 6 and 8 weeks after flowering using published 26 soybean accessions. Asterisk indicates significant correlation (*P* < 0.01, Pearson correlation)

2. The authors acknowledge the existence of numerous GWAS studies on seed traits in soybean, and the major challenge lies in identifying specific genes underlying these traits. Although the study identifies some candidate genes and attempts to verify their functions, the significance of the GWAS-TWAS approach used here is not sufficiently validated. The USPL1 was selected due to its homology to the Arabidopsis, whereas the RWOS1 tends to play quite a minor role in regulating seed traits according to the authors' data. Besides, even they regulate seed traits, natural variations and their contributions to seed traits require further examination.

Response: Genetic variations may affect plant phenotypes by changing protein structure or gene expression. In our study, a total of 22 candidate causal genes for seed traits were further prioritized by TWAS (Table 1), confirming the availability of TWAS in pinpointing causal genes. Furthermore, we identified many candidate genes involved in regulation of seed weight and oil content in module IC79 and IC110 according to correlation between gene expression and seed traits (Figure 4 and Supplemental Figure S9), providing potential targets for molecular breeding of soybean. To confirm the function of *GmRWOS1* on regulation of seed weight and oil content in soybean, we disrupted *GmRWOS1* in Williams 82 via CRISPR/Cas9 genome editing system (Figure 5). Actually, Williams 82 is a soybean variety with high oil content (~21%). However, knocking out *GmRWOS1* still increased oil content

to over 22%, along with a 100-seed weight enhancement of 8.80% (Figure 5). It's worth noting that the minority of germplasms had an oil content exceeding 22% (Supplemental Figure S1D), indicating *GmRWOS1* is a good candidate gene for soybean breeding and improvement.

To evaluate contributions of superior alleles of candidate genes to seed traits, we selected the top 50 and bottom 50 accessions according to seed traits from soybean population, and assessed the aggregation of superior alleles in soybean germplasms. A significant divergency in the number of superior alleles was observed between top and bottom 50 accessions. The accessions exhibiting higher seed weight or oil content carried a greater number of superior alleles (Figure 5A and B). In addition, we observed similar results using another published soybean population with 2,898 accessions (Liu et al., 2020), indicating substantial contributions of these candidate genes to soybean improvement (Supplemental Figure S10, A and B). We added these results in revision.

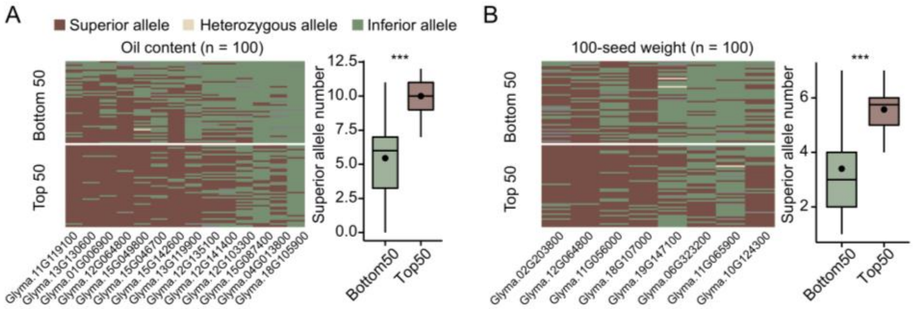

Figure 5, A and B. The aggregation of superior alleles in the top and bottom 50 accessions with highest and lowest oil content (A) or 100-seed weight (B) in the soybean population, respectively.

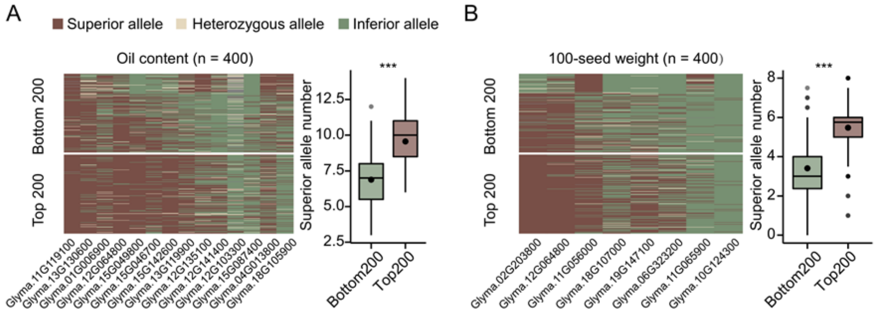

Supplemental Figure S10, A and B. The aggregation of superior alleles in the top and bottom 50 accessions with highest and lowest oil content (A) or 100-seed weight (B) in another

published soybean population, respectively.

3. The selection analysis of RWOS1 might benefit from a whole-genome scale evaluation rather than being limited to the local region, despite this kind of analyses has been widely published in the literature.

Response: Thanks for this suggestion. The F_{ST} value (0.52) across the genomic region flanking the *GmRWOS1* reached the threshold of 0.48 (top 10%) using whole-genome scale evaluation (Figure 6D), although the nucleotide diversity (π) ratio (2.25) between wild and landrace was below the threshold of 2.83 (top 10%). Regarding improvement, the π ratio of landrace relative to cultivar exceed the cutoff of 2.14 (top 10%) (Figure 6D). These results suggest *GmRWOS1* underwent strong artificial selection during domestication and improvement of soybean. The figure and results were updated in revision.

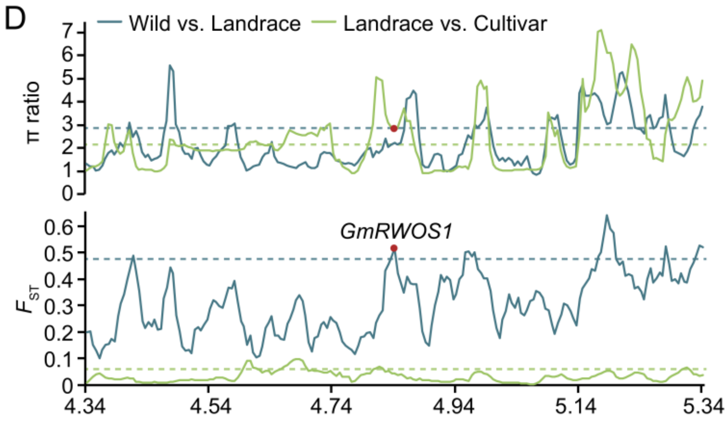

Figure 6D. The π ratio and F_{ST} values of the genomic region flanking the *GmRWOS1* in wild soybeans, landraces and cultivars. The horizontal dashed lines indicate the genome-wide thresholds of wild vs. landrace and landrace vs. cultivar (top 10%). The *GmRWOS1* is labeled by red dot.

Reviewer #2 (Comments for the Author):

Seed weight and oil content are two most important determinants of soybean yield and quality. There is a high correlation between seed weight and oil content. Hundreds

of quantitative trait loci (QTLs) controlling seed weight and oil content have been identified, but the genetic mechanism and causal genes underlying QTLs synergistically regulating seed weight and oil content remain largely unknown. Here, the authors explored genetic loci and identified co-expression modules and candidate genes that significantly associated with seed weight and oil content in soybean by integration of GWAS, expression quantitative trait locus (eQTL) information and TWAS. Candidate causal genes GmRWOS1 and GmUSPL1 were further studied using genome-editing strategy and resulted in synergistical variation in seed weight and oil content. This is an interesting and important work and with clearly presented results that should be of wide interest to Large-Scale Biology community and those interested in breeding of high-yield and high-quality varieties in soybean. I recommend acceptance following satisfactory revision. However, some concerns need to be addressed.

Response: We appreciate the encouraging and constructive comments from this expert reviewer and have addressed the concerns in this revision.

Major comments:

1. How to control false positive signals caused by population structure when performing GWAS? I did not find this in the METHOD.

Response: As suggested, we added more details in the method "The MLMA leave-one-chromosome-out (LOCO) strategy was used to estimate genetic relationship matrix (GRM) from SNPs except those on the chromosome where candidate SNP was located. The GRM was added as random effect to reduce the impact of population structure when performing association analysis based on MLM".

2. Are there any correlations between the phenotype data from two different years? Additionally, what is the level of correlation between seed weight and oil content?

Response: Given that the collected soybean accessions were primarily collected from high-latitude regions, we performed a genome-wide association analysis using phenotype data gathered at Harbin in 2019 and 2020 as suggested by Reviewer 3 in

revision. The significant correlations in seed oil content (0.617) and 100-seed weight (0.883) were observed among these accessions across two independent years (Supplemental Figure S1B). The Best Linear Unbiased Estimator (BLUE) value for each accession was further calculated for each trait and used for GWAS analysis. A positive correlation (0.23) between seed oil content and 100-seed weight was observed among 421 accessions (Supplemental Figure S1D).

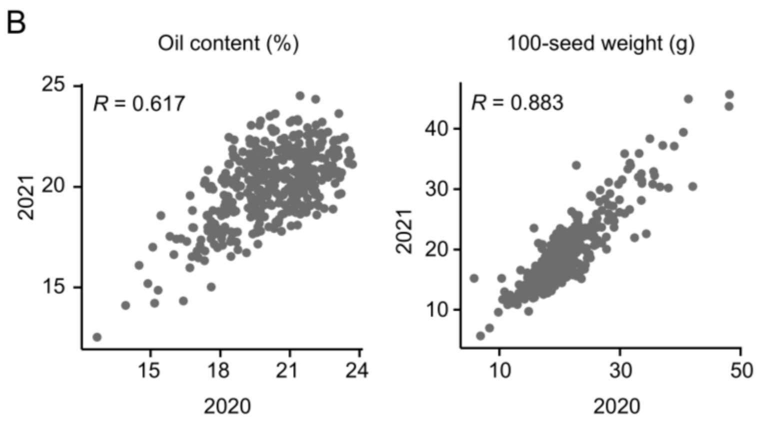

Supplemental Figure S1B. The scatter plots of oil content and 100-seed weight in 2020 and 2021.

3. How were the 238 accessions selected for eQTL analysis? Do they have the same population composition as the 421 accessions? Was the issue of false positive signals caused by population structure considered when performing eGWAS?

Response: We first selected soybean accessions with relatedness (identity-by-state, IBS) < 0.125, and then randomly selected 250 accessions for transcriptome analysis. As we failed to obtained 3’ RNA-seq data from 12 accessions, the transcriptome data of 238 accessions were eventually used for eQTL analysis.

Similar as GWAS, the GRM was added as random effect to reduce the impact of population structure when performing eGWAS.

4. Is there always a positive relationship between seed weight and oil content? It would be helpful to clarify this in the introduction section (page 3:64-67).

Response: In general, seed weight and oil content were positively correlated

(Supplemental Figure S1D). However, some soybean accessions had bigger seeds but lower oil content, or had smaller seeds but higher oil content (Supplemental Figure S1D). We clarified relationship between seed weight and oil content in revision as “In general, seed weight is positively associated with oil content, although negative correlation is also reported in a few soybean germplasms”.

5. In the case of eQTL hotspots, they may regulate multiple genes. However, it's also possible that an "eQTL hotspot" only regulates one upstream gene, such as a transcription factor. In such cases, downstream genes may exhibit similar expression patterns to that of the upstream gene. During eGWAS, these downstream genes might be considered as direct targets of the eQTL hotspot by mistake. How did the authors distinguish between these two situations?

Response: Actually, all eQTL hotspots were distal eQTLs, which indirectly exerted their effects on expressions of eGenes via several mechanisms, primarily through regulation by transcription factors (Yao et al., 2017). As reviewer said, the genetic variations in an eQTL hotspot may affect nearby transcription factor to regulate expressions of downstream genes. Therefore, all regulated eGenes were indirect targets of eQTL hotspots.

6. I am a little bit curious about that the growth condition of GmRWOS1 knockout lines and overexpression lines? I couldn't find this in METHOD. I would also like to see some discussion on GmRWOS1 negatively regulates seed weight and oil content in soybean.

Response: Thanks for this suggestion. We added the growth condition of knockout lines and overexpression lines in METHODS as “The transgenic plants were grown at the experimental station in Nanjing during the summer season in 2022 and 2023”. As suggested, we added some discussion about the function of *GmRWOS1* in DISSCUSSION as “*GmRWOS1* encodes a K-stimulated pyrophosphate-energized sodium pump protein. In animals, the sodium pump serves as the main active transport system, generating a membrane potential and sodium gradient that are

utilized by ion channels and cotransporters. However, the role of the sodium pump in plants remains unclear. Inappropriate sodium concentrations may result in lower seed mass and seed viability (Kranner and Seal, 2013). Molecular mechanism controlling seed weight and oil content by *GmRWOS1* remains to be further studied in future”.

Minor comment:

1. Please clarify the line color used in the FST figure.

Response: Sorry about this mistake and we have corrected the line color in revision.

Reviewer #3 (Comments for the Author):

In this manuscript, Yuan et al dissected the genetic architectures of seed weight and oil content by combining GWAS and TWAS using 421 soybean accessions. They also performed eQTL analysis using the transcriptional data of 238 soybean accessions. The integration of multiple-omics analyses identified and validated GmRWOS1 and GmUSPL1 to be involved in regulation of seed weight and oil content in soybean. It is a very interesting work and the manuscript is well written, which also provides a good resource for soybean functional study, particularly for seed development. However, some points are not clear in the current version, which may need to be further clarified or investigated.

Response: We appreciate the encouraging and constructive comments from this expert reviewer and have addressed the concerns in this revision.

1. The authors collected the soybean accessions from 24 countries in four main regions with similar latitude. Is this true? Based on the map, looks like the accession ranged a lot in the latitudes. Please confirm.

Then, the author phenotype them in three locations, Harbin, Nanjing, Sanya. If the accessions from similar latitude, I am not sure if this kind of design using phenotype from three locations are good or not. As soybean is quite sensitive to environment, I believe the accessions may not grow normally in either two locations of Harbin,

Nanjing, or Sanya as they are from quite different latitude, since the soybeans mainly come from the similar latitude. If the accessions from similar latitude, I prefer using data from the same location from different years, which may be more reasonable.

The authors need to provide the phenotypic divergence among different locations, and also to check the consistence among GWAS results using individual environment data.

Response: Thanks for the valid comments. All accessions were collected from high-latitude regions. We revised the sentence as "We collected 421 cultivated accessions from 24 countries in four main regions with high latitude".

As suggested, we performed GWAS only using two-year phenotypes harvested in Harbin. The significant correlations in seed oil content (0.617) and 100-seed weight (0.883) were observed among these accessions across two independent years (Supplemental Figure S1B). The Best Linear Unbiased Estimator (BLUE) value for each accession was further calculated for each trait and used for GWAS analysis. All related results were updated in revision.

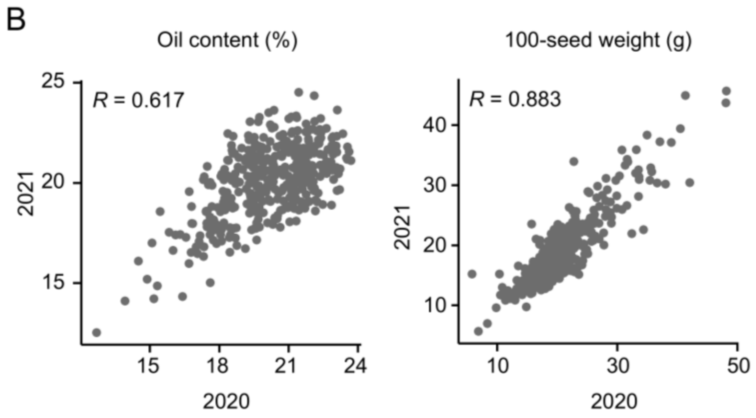

Supplemental Figure S1B. The scatter plots of oil content and 100-seed weight in 2020 and 2021.

2. It is very interesting for the phenotyping results, the authors found North America exhibited the highest oil content, while accessions from East Asia showed the lowest oil content, but highest 100-seed weight. As the authors cited and found, the seed weight and oil content usually showed positive correlation. Why this is not the case for that from the East Asia? Are the accessions showed highest 100-seed weight same

to that showed lowest oil content? This may need to be checked and to give a description. If this is the case, it means that some genes are specifically broke the co-regulation of seed weight and oil content in the East Asia soybean, which is more interesting that deserve to be deeply investigated.

Response: In general, seed weight and oil content were positively correlated (Supplemental Figure S1D). However, some soybean accessions had bigger seeds but lower oil content, or had smaller seeds but higher oil content (Supplemental Figure S1D). The significant peaks identified by GWAS for seed weight and oil content were mostly nonoverlapping, indicating that most major variants controlling the two traits were independent (Supplemental Data Set S3). The accessions in East Asia still exhibited positive relationship between seed weight and oil content (Supplemental Figure S1D). The accessions in East Asia with highest 100-seed weight also showed higher oil content (Supplemental Figure S1D). The lowest median value of oil content in accessions from East Asia was due to higher percentage of accessions with lower oil content (Supplemental Figure S1D). We added these results in revision.

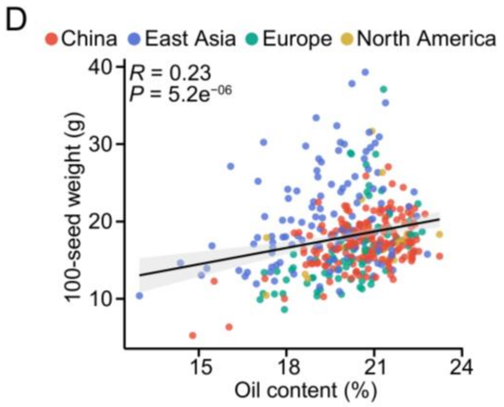

Supplemental Figure S1D. The correlation between oil content and 100-seed weight.

3. Using GWAS, a total of 21 and 13 significantly associated loci were detected for oil content and 100-seed weight, respectively. Following, the authors using TWAS also identified some gene associated with oil content and 100-seed weight, respectively. What is overlap of these two dataset? Are the TWAS genes from individual GWAS regions, or may some come from the same GWAS regions? This data need to be

provided.

Response: Thanks for this suggestion. In total, 50% (7/14) and 37.5% (3/8) of genes identified by TWAS located in significantly associated loci detected by GWAS (Supplemental Data Set S3; Table 1). Most TWAS genes were derived from individual GWAS loci. Only two genes (*Glyma.15G046700* and *Glyma.15G049800*) located in same GWAS locus. We added the related information in Supplemental Data Set S3 in revision.

4. The authors found significant positive genetic correlation between seed weight and oil content, then also found the minor alleles that exceeded significant threshold mainly showed negative effect for oil content, whereas most minor alleles exerted positive influence to seed weight. The results is very interesting. How to explain that the minor alleles showed negative effect for oil content but exerted positive influence to seed weight? Is there any specific cases to show these effects? It will be very interesting to give some examples to show this kind of genes may broke the co-regulation of seed weight and oil content in soybean.

Response: These are valid comments. Actually, most minor alleles regulated seed weight and oil content independently (Supplemental Data Set S3). We only identified two major alleles showing significant association with both seed weight and oil content by GWAS (Supplemental Data Set S3). These two alleles had same direction of effect for seed weight and oil content.

5. For the eQTLs hotspots, I am a little bit confuse, or not clear. The authors found most of these hotspots overlapped with previously reported QTLs and known genes related to seed traits, especially seed weight or oil content. But GO and KEGG enrichment analysis revealed eGenes regulated by distinct eQTL hotspots were enriched in different terms. If most of these hotspots overlapped with previously reported QTLs and known genes related to seed traits, especially seed weight or oil content, the genes regulated by these eQTL hotspots should enriched some terms also related to seed weight or oil content, at least some are similar. However, almost no

overlap for different eQTLs hotspots.

In addition, are the eQTLs all distal eQTLs? Or what is ratio?

Response: In fact, most eQTL hotspots regulated ~20 eGenes, which make it difficult to enrich any GO terms or KEGG pathways (Figure 2F, G). Although 23 eQTL hotspots were identified, we obtained enriched GO terms or KEGG pathways for eGenes regulated by 8 eQTL hotspots. On the other hand, the eQTL hotspots overlapped with QTLs for same phenotype may regulated different pathways to affect the specific phenotype. Actually, different eQTL hotspots regulated distinct genes and very limited eGenes could be regulated by multiple eQTL hotspots (Figure 2G). Therefore, eGenes regulated by distinct eQTL hotspots were enriched in different terms.

All eQTL hotspots were distal eQTLs. We added this information in revision.

6. for figure 3A, I am not quite sure why talk about protein, the whole story did not talk about protein.

In addition, why the top panel only shows 10 eQTLs hotspots, other eQTLs hotspots do not have these kind of pattern/relationship? Or this kind of relationship only randomly associated but did not provide too much clear clues?

Response: As suggested, we removed the information about protein in revision.

For 23 eQTL hotspots, we selected the ten hotspots with the highest number of distally regulated genes for analysis. The other eQTL hotspots regulated fewer genes and had limited relationship with these modules. We clarified these 10 eQTL hotspots in revision.

7. Still Figure 3A, the authors found that two modules (IC79 and IC110) showed significant correlation with both 100-seed weight and oil content, whereas only IC79 (1,357 genes) exhibited identical direction of associations with both traits. I am more interesting in IC110, are the genes in this module only control oil content? Some specific cases?

Response: Many thanks for this suggestion. We further explored the function of

module IC110 in revision. IC110 were subdivided into three submodules. In submodule 1, genes were positively correlated with oil content and negatively correlated with 100-seed weight, while the expression patterns of genes in submodules 2 and 3 were contrast to submodule 1 (Supplemental Figure S9, A and B). Furthermore, we found 30 genes showing opposite correlation with 100-seed weight and oil content (P < 0.01), including *FBA2* and *PME47* (Supplemental Figure S9C). Consistent with this notion, *PME47* was reported to be involved in regulation of seed weight, and *FBA2* was recognized as a candidate gene for oil content in previous studies (Du et al., 2017; Kumar et al., 2022).

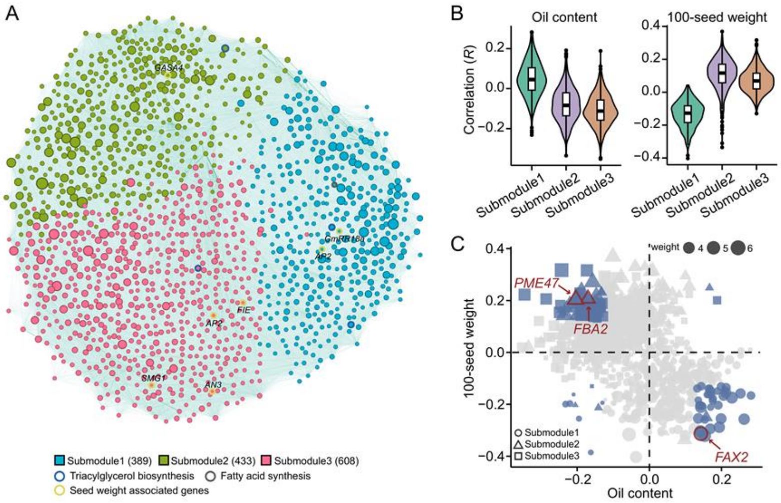

Supplemental Figure S9. A, Correlation network of genes in module IC110. The genes related to seed weight and lipid synthesis are labeled in the network. B, Correlation of genes in different submodules with 100-seed weight (right) and oil content (left). C, The scatter plot of correlation of genes in three submodules with 100-seed weight (y-axis) and oil content (x-axis). The genes significantly associated with both 100-seed weight and oil content are marked in blue ($P < 0.01$, Pearson correlation).

8. The author found that Glyma.11G119100 showed positive correlation with both seed traits and was colocalized with loci significantly associated with oil content in GWAS as well. What is the causal genetic variation for this gene, which needs to be provided? In addition the authors only provided knock out line. Strictly, at least OE

lines are also needed.

Response: Three major haplotypes of *GmUSPL1* were identified, of which 314 accessions harbored Hap 1, 49 accessions harbored Hap 2 and 30 accessions harbored Hap 3 (Supplemental Figure S6A). Accessions with Hap 3 showed lower oil content and reduced expression of *GmUSPL1* compared to accessions harboring Hap 1/2 (Supplemental Figure S6B). We added these results in revision.

To further confirm function of *GmUSPL1*, we obtained another homozygous knockout line for *GmUSPL1* by CRISPR/Cas9 (Supplemental Figure S6C). We obtained phenotypes of two knockout lines (CR1 and CR2) of *GmUSPL1* in 2023. Compared to the wild type, both knockout lines exhibited decrease of oil content and 100-seed weight (Figure 4, B and C), confirming the function of *GmUSPL1* in regulation of seed weight and oil content.

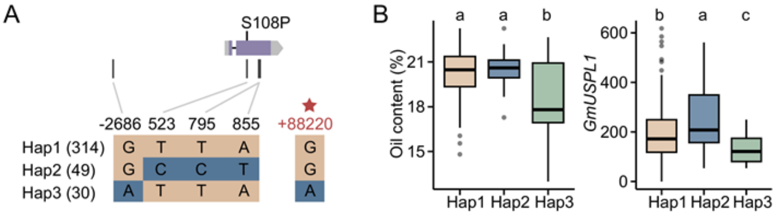

Supplemental Figure S6. A, The spectrum of three haplotypes for *GmUSPL1*. Vertical lines represent variants. The lead SNP for oil content is marked in red star. **B,** Differences of oil content (left) and *GmUSPL1* expression (right) between individuals with different haplotypes. A one-way ANOVA and SNK test were used for statistical analysis.

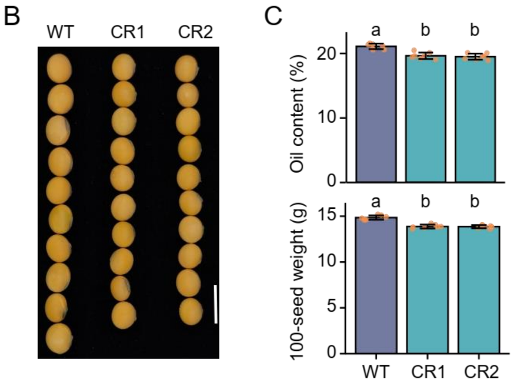

Figure 4. B, The representative seeds of wild-type plant and *gmuspl1* mutants (CR1 and CR2). Scale bar, 1 cm. C, Seed oil content (upper) and 100-seed weight (bottom) in wild type and *gmuspl1* mutants. Two-tailed t-test was used for statistical analysis. The value of each

replicate is represented by a dot.

9. For Supplemental Figure S7, Haplotype analysis of MYC2, GmBZR1 and bZIP44, the expression did not show significant differences as the phenotype, which is not so consistent with the claim of the correlation between expression differentiation and phenotype changes.

Response: There were significant differences in 100-seed weight, oil content and IC79 expression levels among accessions with distinct haplotypes for *MYC2* (Hap1 vs Hap2/3) and *bZIP44* (Hap1 vs Hap2) (Supplemental Figure S8), which was consistent with the claim of negative correlation between IC79 expression and seed traits. The divergence of seed traits and IC79 expression was not significant among haplotypes of *GmBZR1*. Therefore, *MYC2* and *bZIP44* may be the potential regulators of module IC79. We added this information in revision.

References

Du, J., Wang, S., He, C., Zhou, B., Ruan, Y.L., and Shou, H. (2017). Identification of regulatory networks and hub genes controlling soybean seed set and size using RNA sequencing analysis. J Exp Bot **68,** 1955-1972.

Kranner, I., and Seal, C.E. (2013). Salt stress, signalling and redox control in seeds. Funct Plant Biol **40,** 848-859.

Kumar, V., Goyal, V., Mandlik, R., Kumawat, S., Sudhakaran, S., Padalkar, G., Rana, N., Deshmukh, R., Roy, J., Sharma, T.R., and Sonah, H. (2022). Pinpointing Genomic Regions and Candidate Genes Associated with Seed Oil and Protein Content in Soybean through an Integrative Transcriptomic and QTL Meta-Analysis. Cells **12**.

Liu, Y., Du, H., Li, P., Shen, Y., Peng, H., Liu, S., Zhou, G.A., Zhang, H., Liu, Z., Shi, M., Huang, X., Li, Y., Zhang, M., Wang, Z., Zhu, B., Han, B., Liang, C., and Tian, Z. (2020). Pan-Genome of Wild and Cultivated Soybeans. Cell **182,** 162-176 e113.

Yao, C., Joehanes, R., Johnson, A.D., Huan, T., Liu, C., Freedman, J.E., Munson,

P.J., Hill, D.E., Vidal, M., and Levy, D. (2017). Dynamic Role of trans Regulation of Gene Expression in Relation to Complex Traits. Am J Hum Genet **100,** 571-580.

1st Revision - Editorial Decision20-Jan-2024

Dear Dr. Song:

We have received reviews of your manuscript entitled "Integrative omics analysis elucidates the genetic basis underlying synergistic regulation of seed weight and oil content in soybean." On the basis of the advice received, the board of reviewing editors would like to accept your manuscript for publication in The Plant Cell. This acceptance is contingent on revision based on the comments of our reviewers.

Please consider the comments of reviewer 1, about the title. For example, as an alternative, a new title could be "Integrative omics analysis elucidates the genetic basis underlying genetic regulation of seed weight and oil content in soybean".

Please highlight all changes and include a detailed annotation of changes of the text, with line numbers, and noting your responses to the comments.

To submit your revised manuscript, click:

Link Not Available

If you have any questions about the revision submission procedures, please contact the Editorial Office Staff (tpc-submissions@aspb.org). If you cannot return the revised manuscript within 30 days, please let us know. Otherwise, we will assume that you have elected not to revise the manuscript and withdraw it.

Thank you very much for the privilege of reviewing this work. I look forward to receiving the next version.

On behalf of the editorial board,

Jinsheng Lai, Board of Reviewing Editors
James Birchler, Senior Editor
Blake Meyers, Editor-in-Chief

The Plant Cell

Reviewer #1 (Comments for the Author):

While I am mostly satisfied with the authors' responses, one remained concern is regarding the synergistic regulation that the authors used in the title. In this study, it appears that the authors use the word to describe the simultaneous regulation of two traits by one gene or module. Could this really be described as synergistic regulation? Please confirm.

Reviewer #2 (Comments for the Author):

Minor comment:
243 and 246, Change GmUSPL1 to Glyma.11G119100

Reviewer #3 (Comments for the Author):

Thank the authors for their constructive revise. All my questions were addressed. I do not have further comments.

Please also note the following:

-The Plant Cell now requires authors to complete and submit an author revisions checklist upon submission of a revised manuscript. The aim of the checklist is to aid authors in preparing a high-quality manuscript, facilitate the review and assessment of revised manuscripts, and help to ensure that journal standards are maintained across the board. If your manuscript is accepted, the completed checklist will be published as supplemental material attached to the article online. Please

download a copy of the checklist (pdf fillable form) at this link, for submission with your revised manuscript: https://tpc.msubmit.net/html/Author_Revisions_Checklist.pdf.

-Supplemental materials should be restricted to large datasets and tables, presentation of replicates, and validation of reagents, methods, or genotypes. Any data that are used to support the major claims must be in the main manuscript. Supplemental figure legends must indicate what figure in the main manuscript is supported by the supplemental data presented. Please justify how each of the supplemental figures meet the criteria.

-Sampling methods and the nature of "biological replicates" should be described precisely (i.e. different plants, parts of plants, pooled tissue, independent pools of tissue, sampled at different times, etc.), along with a clear description of and rationale for any statistical analyses conducted. The reader should know exactly what was sampled; what forms the basis of the calculation of any means and statistical parameters reported. This is also necessary to ensure that proper statistical analysis was conducted.

Reviewer #1 (Comments for the Author):

While I am mostly satisfied with the authors' responses, one remained concern is regarding the synergistic regulation that the authors used in the title. In this study, it appears that the authors use the word to describe the simultaneous regulation of two traits by one gene or module. Could this really be described as synergistic regulation? Please confirm.

Response: To exclude potential misinterpretation, we changed the title to “Integrative omics analysis elucidates the genetic basis underlying regulation of seed weight and oil content in soybean” in revision. We appreciate suggestions from this expert reviewer which are very helpful for improving our manuscript.

Reviewer #2 (Comments for the Author):

Minor comment:

243 and 246, Change GmUSPL1 to Glyma.11G119100.

Response: We changed ‘*GmUSPL1*’ to ‘*Glyma.11G119100*’ in line 243 and 246 in revision. We appreciate suggestions from this expert reviewer which are very helpful for improving our manuscript.

Reviewer #3 (Comments for the Author):

Thank the authors for their constructive revise. All my questions were addressed. I do not have further comments.

Response: We appreciate suggestions from this expert reviewer which are very helpful for improving our manuscript.

Dear Dr. Qingxin Song:

We are pleased to inform you that your paper entitled "Integrative omics analysis elucidates the genetic basis underlying regulation of seed weight and oil content in soybean" has been accepted for publication in The Plant Cell, pending a final editorial review by a science editor. At this stage, your manuscript will be evaluated by a science editor with respect to its presentation of scientific content, compliance with journal policies, and presentation for a broad readership. The Plant Cell has contracted with Plant Editors (planteditors.com) to provide this service to our authors, and you will soon receive additional information on this process.

Please note that each author needs to link their ORCID identifier to their account in the system before your manuscript can be published. If any authors do not have an ORCID linked to their account, they will receive a message with a link to complete this task. Please ensure that ALL of your coauthors have completed this task as soon as possible.

ASPB offers an OPEN option that allows authors to have their online articles available for free to all users immediately upon publication. For more information about the ASPB OPEN option, refer to the Final Submission Checklist Form.

The Plant Cell and The Arabidopsis Information Resource (TAIR) are collaborating to collect functional annotation data about Arabidopsis genes from authors. This includes information about the gene's molecular function (e.g., kinase activity, ATP synthetase activity), the biological process/es it is involved in (e.g., endosperm development, threonine biosynthesis), its subcellular location (e.g., nucleus, ER), anatomical or developmental expression pattern (e.g., leaf, ovule, flower stage 10, seedling stage), or its partner in a protein-protein interaction (e.g., AT1G01010 interacts with AT1G01020).

If your paper contains results falling into one or more of these categories for Arabidopsis genes, we request that you now submit these data for inclusion in TAIR by filling in the form provided at the following URL: https://www.arabidopsis.org/doc/submit/functional_annotation/123. If you need further clarification on what types of data can be submitted please contact curator@arabidopsis.org.

Finally, we encourage your submission of artwork for the journal cover. Monthly issues will have an online cover image and selected covers will be used for posters, other promotional items, and "wallpaper" for mobile devices. For more information, refer to Cover Submission in the Instructions for Authors https://tpc.msubmit.net/cgi-bin/main.plex?form_type=display_auth_instructions.

We look forward to seeing your paper published.

On behalf of the editorial board,

Reviewing Editor, Board of Reviewing Editors
James Birchler, Senior Editor
Blake Meyers, Editor-in-Chief

The Plant Cell

=====
IMPORTANT REMINDER: PEER REVIEW REPORTS
=====

If you opted to publish a peer review report along with your article during the original submission process, it will be prepared by the editorial staff and publicly posted with your manuscript, inside the zip file that contains any other supplemental material. As a reminder, the peer review report is a public record of all comments from editors and reviewers, as well as your prior responses, as you received them in the decision letters for each draft of your manuscript. If you agreed to publish this report and have changed your mind, or are not sure if you selected this option, please contact the editorial office as soon as possible before signing the license agreement from our publisher.

=====

----FOR ASPB OFFICE USE ONLY (DO NOT EDIT)----
MSID: 37674
Corresponding Author: Dr. Song
